# The cAMP pathway is important for controlling the morphological switch to the pathogenic yeast form of *Paracoccidioides brasiliensis*

**DOI:** 10.1111/j.1365-2958.2007.05824.x

**Published:** 2007-08-01

**Authors:** Daliang Chen, Thamarai K Janganan, Gongyou Chen, Everaldo R Marques, Marcia R Kress, Gustavo H Goldman, Adrian R Walmsley, M Inês Borges-Walmsley

**Affiliations:** 1 Centre for Infectious Diseases, Wolfson Research Institute, School of Biological and Biomedical Sciences, University of Durham – Queen's Campus Stockton-on-Tees TS17 6BH, UK.; 2 Departamento de Ciencias Farmaceuticas, Faculdade de Ciencias Farmaceuticas de Ribeirao Preto, Universidade de Sao Paulo, Av. do Cafe S/N CEP 14040-903, Ribeirao Preto, Sao Paulo, Brazil.

## Abstract

*Paracoccidioides brasiliensis* is a human pathogenic fungus that switches from a saprobic mycelium to a pathogenic yeast. Consistent with the morphological transition being regulated by the cAMP-signalling pathway, there is an increase in cellular cAMP levels both transiently at the onset (< 24 h) and progressively in the later stages (> 120 h) of the transition to the yeast form, and this transition can be modulated by exogenous cAMP. We have cloned the *cyr1* gene encoding adenylate cyclase (AC) and established that its transcript levels correlate with cAMP levels. In addition, we have cloned the genes encoding three Gα (Gpa1–3), Gβ (Gpb1) and Gγ (Gpg1) G proteins. Gpa1 and Gpb1 interact with one another and the N-terminus of AC, but neither Gpa2 nor Gpa3 interacted with Gpb1 or AC. The interaction of Gpa1 with Gpb1 was blocked by GTP, but its interaction with AC was independent of bound nucleotide. The transcript levels for *gpa1*, *gpb1* and *gpg1* were similar in mycelium, but there was a transient excess of *gpb1* during the transition, and an excess of *gpa1* in yeast. We have interpreted our findings in terms of a novel signalling mechanism in which the activity of AC is differentially modulated by Gpa1 and Gpb1 to maintain the signal over the 10 days needed for the morphological switch.

## Introduction

The phylogenetically related ascomycete fungi *Paracoccidioides brasiliensis*, *Histoplasma capsulatum*, *Blastomyces dermatitidis*, *Coccidioides immitis*, *Penicillium marneffei* and *Sporothrix schenkii* from more than hundred thousand different species of environmental fungi are able to adapt for survival in mammalian hosts (Borges-Walmsley *et al*., 2002; Morris-Jones, 2002; San-Blas *et al*., 2002; Bradsher *et al*., 2003; DiCaudo, 2006; Vanittanakom *et al*., 2006; Kauffman, 2007). These are known as dimorphic fungi because they undergo extensive changes that allow them to switch from mycelium, a non-pathogenic filamentous form, to pathogenic single-cellular yeast that causes infections in millions of people across the globe every year. Infection is the result of the release from mycelium, frequently found in soil, of fragments or spores, which are inhaled by the host, exposing them to an elevated temperature that triggers the morphological switch. The pathogenicity of these fungi is intimately linked to the morphological change because strains that are unable to transform from mycelium to yeast are often avirulent (Nemecek *et al*., 2006). However, our knowledge of how these fungi sense and respond to the temperature change is still rudimentary.

In eukaryotes, many cell-signalling processes are mediated by guanine-nucleotide binding proteins known as G proteins (for a review, see Sprang, 1997). Generally these are activated when they interact with a G protein-coupled receptor (GPCR) and transmit signals to downstream effectors such as adenylate cyclase (AC) and protein kinases. Typically these G proteins function as heterotrimeric complexes, composed of Gα, Gβ and Gγ subunits, which are activated when GTP binds to, and replaces bound GDP on, the Gα subunit to cause its dissociation from the Gβγ dimer. The signal can be mediated by Gα-GTP, Gβγ or both, depending on the pathway, and is attenuated as the GTP is hydrolysed to allow the re-association of the trimeric complex (Sprang, 1997). Although this is a generally held view of the function of heterotrimeric G proteins, there is recent evidence that complex dissociation is not needed for signalling by all G proteins (Frank *et al*., 2005).

Fungi possess between two and four, but most have three, Gα proteins. However, they only appear to have a single Gβ and Gγ protein, suggesting that either some Gα proteins can act independently or multiple Gα proteins can interact with the Gβγ dimer (Lafon *et al*., 2006; Yu, 2006). *Saccharomyces cerevisiae* has two Gα proteins, Gpa1 and Gpa2, which have been shown to regulate mating, in response to pheromones, and filamentous growth, in response to glucose, via mitogen-activated protein kinase (MAPK) and cAMP-signalling pathways respectively (for a recent review, see Hoffman, 2005). Gpa1 interacts with the Gβγ dimer, composed of Ste4 and Ste18, preventing activation of the MAPK-signalling pathway by the Ste4–Ste18 dimer, which binds to the scaffold protein Ste5 (Whiteway *et al*., 1995; Pryciak and Huntress, 1998) and the p21-activated kinase Ste20 (Leeuw *et al*., 1998). In contrast, the cAMP-signalling pathway is activated by Gpa2 and Ras (Lorenz and Heitman, 1997; Colombo *et al*., 1998; 2004; Xue *et al*., 1998; Kraakman *et al*., 1999; Lorenz *et al*., 2000), both of which are presumed to bind directly to AC. Although Gpa2 does not appear to have a Gβ partner, it can bind the kelch-repeat proteins Gpb1 and Gpb2 that may mimic Gβ subunits to control the level of the free protein (Harashim and Heitman, 2002). *Schizosaccharomyces pombe* also possesses two Gα proteins that regulate pheromone-activated MAPK and glucose-activated cAMP-signalling pathways. However, the pheromone pathway is activated by the Gα protein Gpa1, rather than by a Gβγ dimer, but its target is unknown (Obara *et al*., 1991; Ladds *et al*., 2005); while Gpa2, which binds to the Gβγ dimer, composed of Git5 and Git11 (Landry and Hoffman, 2001), binds to and activates AC (Ogihara *et al*., 2004; Ivey and Hoffman, 2005). *Ustilago maydis* has four Gα proteins, but it is not known whether they work independently of a Gβ or whether they all interact with the same Gβ, or even if they have kelch-repeat protein partners (Regenfelder *et al*., 1997; Muller *et al*., 2004). *Aspergillus nidulans* possesses three Gα proteins, FadA (Yu *et al.*, 1996), GanA and GanB (Chang *et al*., 2004), and apparently a single Gβ, sfaD (Rosen *et al.*, 1999), and Gγ, GpgA (Seo *et al.*, 2005), protein, but does not appear to possess kelch-repeat proteins that are related to Gpb1 or Gpb2. There is evidence from the phenotypes of disruption mutants that a sfaD–GpgA dimer can interact with both FadA and GanB (Lafon *et al*., 2005), but a direct interaction has not been demonstrated.

Gβ proteins have been found to be involved in developmental pathways of several filamentous fungi: for example, in controlling the development and/or virulence of the plant pathogens *Cryphonectria parasitica* (Kasahara and Nuss, 1997), *Magnaporthe grisea* (Nishimura *et al*., 2003), *Fusarium oxysporum* (Jain *et al.*, 2003; Delgado-Jarana *et al*., 2005), *U. maydis* (Muller *et al*., 2004) and *Cochliobolus heterostrophus* (Ganem *et al.*, 2004), and in the development of *Neurospora crassa* (Krystofova and Borkovich, 2005), *A. nidulans* (Rosen *et al*., 1999) and *Cryptococcus neoformans* (Wang *et al.*, 2000). Although the Gβ proteins MBP1 and GPB1, from *M. grisea* and *C. neoformans*, appear to function through MAPK-signalling pathways in analogy with Ste4, the other Gβ proteins function, at least partially, through cAMP-signalling pathways. In several cases, exogenous cAMP can suppress some, if not all, of the defects caused by deleting these genes. For example, the filamentous growth of a *bpp1* deletion in *U. maydis* can be suppressed by exogenous cAMP (Muller *et al*., 2004). A constitutively active allele of *gpa3* also suppresses this phenotype, suggesting that Bpp1 and Gpa3 are components of the same heterotrimeric G protein that acts on AC. However, in contrast to Δ*bbp1* strains, Δ*gpa3* strains are impaired in pathogenicity, suggesting that Gpa3 operates independently of Bpp1. In *N. crassa*, deletion of the Gβ protein Gnb-1 causes a reduction in cAMP levels, but this was attributed to a reduction in Gα proteins that activate AC (Krystofova and Borkovich, 2005). In *S. pombe*, deletion of the Gβ protein Git5 does not affect basal cAMP levels but inhibits the glucose-induced elevation of cAMP levels (Landry *et al*., 2000), and one proposal is that Git5 interacts directly with AC. Thus, there is evidence that Gα and Gβ proteins can independently activate different signalling processes.

If Gα and Gβ, and the Gβγ and Gαβγ complexes can all elicit signalling, then there is no reason why these subunits should be expressed equivalently. Signalling could be brought about by the increased expression of either the Gα or Gβ subunit, above that of its cognate subunit, so that there would be a pool of uncomplexed G protein that would be free to interact with other proteins in the signalling pathway. In the case of *S. cerevisiae*, it has been reported that there is an excess of Gpa1 over Ste4 (Ghaemmaghami *et al*., 2003) that might be needed to prevent pheromone-independent signalling by the Ste4–Ste18 dimer (Hoffman, 2005). This balance in the subunits is important because a twofold increase in Ste4 is sufficient to activate the pathway (Hao *et al*., 2003). A similar imbalance has been noted in *S. pombe*, where the Gβ Git5 is transcribed at much lower levels than the Gα Gpa2 and Gγ Git11 (Hoffman, 2005). It has been hypothesized that the uncomplexed Gpa2 is associated with AC to form an inactive complex: glucose activation of the signalling pathway would lead to Gpa2-GTP, released from the Git5–Git11 dimer, being swapped for Gpa2-GDP bound to AC that would then become activated (Hoffman, 2005).

The cAMP-signalling pathway has been shown to be important in controlling morphological changes and the pathogenicity of several fungi (Borges-Walmsley and Walmsley, 2000). For example, signalling through AC controls the virulence of *Candida albicans* (Rocha *et al*., 2001), *C. neoformans* (Alspaugh *et al.*, 2002) and *A. fumigatus* (Liebmann *et al.*, 2003). In contrast to the ACs of *C. neoformans* (Vallim *et al.*, 2005) and *A. fumigatus* (Liebmann *et al.*, 2003) that only appear to be regulated via Gα proteins, *C. albicans* is regulated by both Ras (Rocha *et al*., 2001) and Gpa2 (Miwa *et al*., 2004; Maidan *et al*., 2005), which can also interact with the MAPK pathway (Sanchez-Martinez and Perez-Martin, 2002; Bennett and Johnson, 2006). In the plant pathogen *M. grisea*, the morphological changes that are involved in pathogenicity are dependent upon G protein-mediated cAMP signalling, and exogenous cAMP induces formation of the infective appressorium (Nishimura *et al*., 2003).

*Paracoccidioides brasiliensis*, the aetiological agent of paracoccidioidomycosis (for a review, see San-Blas *et al*., 2002; Borges-Walmsley *et al*., 2002), the most prevalent systemic mycosis in Latin America, where it is estimated that throughout the endemic region as many as 10 million individuals, out of a population of about 90 million, may be infected (Restrepo *et al*., 2001). The fungus is dimorphic undergoing a complex transformation *in vivo*, in which mycelia and conidia transform to the pathogenic yeast form (Medoff *et al*., 1987; Borges-Walmsley *et al*., 2002; San-Blas *et al*., 2002), while strains that are unable to undergo the mycelium-to-yeast transformation are avirulent (San-Blas and Niño-Veja, 2001). Herein we establish that this process is regulated by the cAMP-signalling pathway, and we have cloned the gene encoding AC and genes that encode a set of G proteins, which potentially function upstream of AC. In the absence of molecular tools for forward and reverse genetic approaches for studying gene function in *P. brasiliensis*, we have investigated the interaction of these proteins using yeast two-hybrid and pull-down assays. Our data indicate that the Gβ and Gγ proteins Gpb1 and Gpg1 interact specifically with the Gα Gpa1, but not with Gpa2 or Gpa3, to form a trimeric complex. This trimer can dissociate to release Gpa1 and Gpb1 that can independently interact with the N-terminus of AC. We propose that the ability of AC to bind both Gpa1 and Gpb1 enables maintenance of a long-term signal that is required to direct the morphological transition from the saprobic mycelium to pathogenic yeast, which occurs over a period of about 10 days.

## Results

### The *P. brasiliensis* morphological transition is controlled by cAMP

**Fig. 1:**
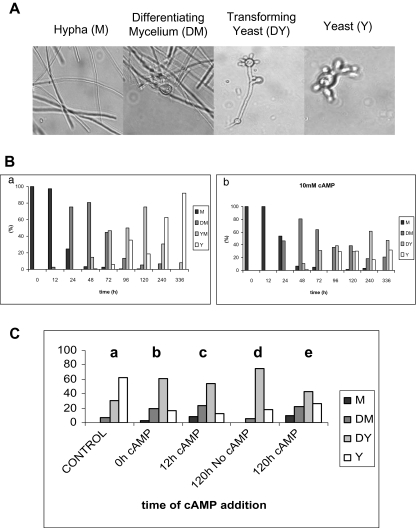
The cAMP-signalling pathway regulates the transition from mycelium to yeast in *P. brasiliensis* A. The morphology of *P. brasiliensis* cells, growing in liquid culture, which had been induced to undergo the mycelium-to-yeast transition by increasing the temperature from 26°C to 37°C and was monitored to quantify the different morphotypes that are produced during this process. Cellular forms were classified into four different morphological states: (i) hyphae; (ii) differentiating hyphae, characterized by the development of chlamydospore-like cells, produced by intercalary or lateral swellings in the fertile hyphae; (iii) transforming yeast, characterized by the production of multiple buds by the chlamydospore; and (iv) mature, multibudding yeast. At the indicated times during the morphological switch, 300 morphological units were picked and the number of individual forms quantified. B. The bar charts show the percentage of each morphological form at increasing times during the morphological transition from mycelium to yeast in the absence (a) and presence (b) of 10 mM dibutyryl-cAMP. The data indicate that exogenous dibutyryl-cAMP retards the mycelium-to-yeast morphological transition. C. The bar charts show the percentage of each morphological form at 240 h after initiating the transition in the absence of cAMP (chart a) and for cells to which 10 mM dibutyryl-cAMP was added at the start of the transition (chart b), at 12 h (chart c) and 120 h (chart e) after initiating the transition; for comparison, the percentage of each morphological forms after 120 h is also shown (chart d). The data indicate that the addition of exogenous dibutyryl-cAMP late in the transition reverses the mycelium-to-yeast morphological transition. M-mycelium; DM-Differentiating mycelium; DY-Differentiating yeast; Y-Yeast.

The cAMP-signalling pathway has been implicated in controlling morphological changes and the virulence of a number of fungi. We sought to determine whether cAMP would affect the morphological transition, which underlies the virulence, of *P. brasiliensis*. As a prerequisite for such an analysis, we monitored the morphology of *P. brasiliensis* cells, growing in liquid culture, which had been induced to undergo the mycelium-to-yeast transition by increasing the temperature from 26°C to 37°C, and established parameters to quantify the different morphotypes that are produced during this process. We classified the transition into four different morphological states (Fig. 1A): (i) hyphae; (ii) differentiating hyphae, characterized by the development of chlamydospore-like cells, produced by intercalary or lateral swellings in the fertile hyphae; (iii) transforming yeast, characterized by the production of multiple buds by the chlamydospore; and (iv) mature, multibudding yeast. This classification helped us establish quantitative parameters to assess the morphological transition at successive time point: after 336 h, 92% of the cells were yeast, indicating that the transition had gone to near completion (Fig. 1B, left graphic). In contrast, when the mycelial cells were treated with 10 mM dibutyryl-cAMP and the morphological switch induced, only 32% of the cells were yeast after 336 h, indicating that cAMP retarded the morphological transition (Fig. 1B, right graphic). We did not find any appreciable effect of dibutyryl-cAMP, at concentrations up to 20 mM, upon the yeast-to-mycelium transition induced by decreasing the incubation temperature from 37°C to 26°C (data not shown). This latter finding contrasts with an earlier study that indicated that cAMP retarded the yeast-to-mycelium transition (Paris and Duran, 1985), but we cannot identify any clear reason for this difference.

### Identification of the components of a cAMP-signalling pathway in *P. brasiliensis*

In an attempt to identify genes from the cAMP-signalling pathway, which our studies clearly implicated in the control of the morphological switch from the mycelium to pathogenic yeast form of *P. brasiliensis*, we used homology-based strategies to clone the genes that encode AC (e.g. *CYR1*) and several G proteins, including three Gα (e.g. *GPA1*, *GPA2* and *GPA3*), Gβ (e.g. *GPB1*) and Gγ (e.g. *GPG1*) subunits, which might be expected to be involved in its regulation (see *Supplementary material*).

**Fig. 2:**
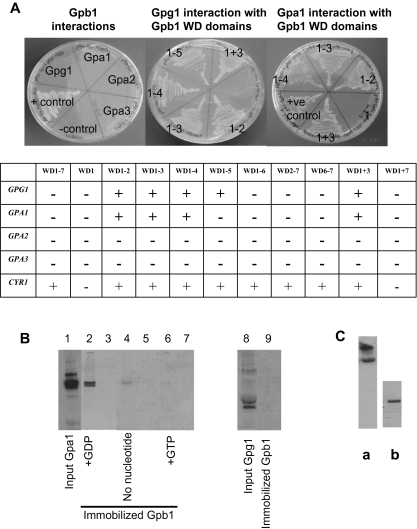
A. *GPA1*, but not *GPA2* nor *GPA3*, and *GPG1* interact with *GPB1*. Each of the *P. brasiliensis* proteins Gpa1, Gpa2, Gpa3, Gpg1 and Cyr1 was tested by two-hybrid screening in *S. cerevisiae* for interactions with Gpb1 (e.g. WD1–7) and a series of truncates in which successive WD domains were deleted from the C-terminus (e.g. WD1–6 to WD1); a construct that lacked the N-terminal WD domain (e.g. WD2–7); a construct that comprised the two C-terminal WD domains (e.g. WD6–7); and a fusion of WD domains 1 and 3 (e.g. WD1+3), and 1 and 7 (e.g. WD1+7). *S. cerevisiae* strain AH109, harbouring pGADT7 plasmids bearing genes that encoded proteins to test for interactions with Gpb1 and truncates of this protein, expressed from pGBKT7, were identified by auxotrophic selection on SD/–Leu/–Trp/–His/–ADE plates and Xgal assays. As illustrated by the left-hand plate, full-length Gpb1 did not interact with Gpa1, Gpa2, Gpa3 nor Gpg1. However, full-length Gpb1 did interact with the positive control Cyr^1−678^, consequently, establishing that the full-length protein is expressed. The middle- and right-hand plates show the interaction of Gpg1 and Gpa1 respectively with the WD domains of Gpb1. As a negative control, each pGBK protein vector was cotransformed, with pGADT7, into AH109 and screen for growth on SD/–Leu/–Trp/–His/–ADE plates. None of these control cells grew (data not shown). In the accompanying table, the (+) and (–) symbols are indicative of the presence and absence of protein–protein interactions respectively. B. Pull-down assays to demonstrate that Gpa1 interacts with Gpb1. GST and GST-Gpb1 were purified from bacteria, loaded onto glutathione sepharose beads before incubation with *in vitro* translated ^35^S-Gpa1and 10 mM nucleotide. After washing the beads, the proteins were eluted by the addition of 4× NuPAGE LDS sample buffer, followed by boiling at 90°C for 5 min, and separated on a 4–12% NuPAGE gel under denaturing conditions. Bound Gpa1 was detected as a gel band by autoradiography. Lanes 2, 4 and 6 establish that Gpa1 binds to immobilized Gpb1, but the apparent affinity decreases in order of incubation with GDP (lane 2), no nucleotide (lane 4) and GTP (lane 6). Negative controls, using immobilized GST, are shown in lanes 3, 5, 7 and 10. Using *in vitro* translated ^35^S-Gpg1 (lane 8), there was no detectable interaction with Gpb1 (lane 9). C. Gpb1 and Gpa1 used in pull-down assays cross-react with specific antibodies. (a) Gpb1 produced as a fusion protein with GST in *E. coli* and (b) Gpa1 synthesized using an *in vitro* transcription/translation system ran at the expected Mr on SDS-PAGE gels and cross-reacted with antibodies generated to specific sequences within these proteins.

A phylogenetic analysis of all known fungal Gα proteins identified to date indicates that they fall into three major families (groups 1–3), each of which is represented by one of the three Gα proteins, Gpa1–3, from *P. brasiliensis* (Fig. S1). In contrast, only a single Gβ and Gγ have been identified in these fungi, raising the question as to whether they interact with more than one Gα. Consequently, we sought to test whether the Gβ protein Gpb1 interacts with Gpa1–3 from *P. brasiliensis* by two-hybrid screening in *S. cerevisiae*. Initially, no interactions between any of the Gα proteins and Gpb1 were detected (Fig. 2A). Although the Gβ protein Ste4 can interact independently, of its cognate Gγ protein Ste18, with the Gα protein Gpa1 in two-hybrid assays in *S. cerevisiae* (Ongay-Larios *et al.*, 2000), it is possible that the failure to detect any interaction between the *P. brasiliensis* Gβ and Gα proteins was attributable to the requirement for a Gγ protein to stabilize Gpb1 for interaction with the Gα proteins. However, we failed to identify an interaction between Gpa1–3 and a Gpb1-linker–Gpg1 fusion protein, nor was there an interaction between Gpb1 and Gpg1 (data not shown). Consequently, we decided to test whether any of the Gpa proteins would interact with discrete domains of Gpb1, which might only be available for interaction in the Gpb1–Gpg1 complex. These experiments revealed an interaction of Gpa1, but not Gpa2 or Gpa3, with C-terminal-truncated Gpb1, with a deletion analysis indicated that Gpa1 interacted with the first two WD domains at the N-terminus (Fig. 2A). Gpa1 also interacted with a fusion protein in which the first WD domain was fused to the third, but not the seventh, WD domain (Fig. 2A). In analogy, *S. cerevisiae* Gpa2 has been reported to interact with an N-terminal-truncated, consisting of residues 531–740 of the, but not with the full-size, kelch-repeat protein Gpb1 (Batlle *et al*., 2003). We complemented this approach by constructing random mutagenesis libraries for Gpa1–3 and Gpb1 in the yeast two-hybrid vector pGADT7: screening the Gpa1–3 libraries with Gpb1 and the Gpb1 library with Gpa1–3. No point or frameshift mutations were detected that led to an interaction (data not shown). As a positive control we used the *GPR1* and *GPA2* genes that, respectively, encode a GPCR and its cognate Gα protein in *S. cerevisiae*: two-hybrid screens indicated that Gpa2 could interact with the C-terminal domain, encompassing residues 679–961, of Gpr1 (data not shown). When a random mutagenesis library of *GPR1*^679−7961^ was screened with *GPA2*, at least 50 positive colonies were detected. Screening the same library with *P. brasiliensis GPA1–3* did not yield any positive colonies, nor did a screen of the *P. brasiliensis GPA1–3* random mutagenesis libraries with *S. cerevisiae GPR1* (data not shown). These results establish that there is a high degree of stringency in the Gα–GPCR interaction because none of the Gα proteins from *P. brasiliensis* could interact with *S. cerevisiae* Gpr1. We conclude that of the three Gα proteins found in *P. brasiliensis*, only Gpa1 interacts with Gpb1, and that it binds to the same N-terminal region of Gpb1 to which Gpg1 binds. These results suggest that Gpa1, Gpb1 and Gpg1 form a Gαβγ trimer.

We used pull-down assays to confirm the interaction of Gpa1, produced by *in vitro* translation, with Gpb1, expressed and purified as a glutathione S-transferase (GST) fusion protein from bacteria. We found that Gpa1 could interact directly with Gpb1, but this interaction appeared to be strengthened by GDP and blocked by GTP (Fig. 2B). The apparent capability of Gpa1 to bind GTP and dissociate from Gpb1 provides strong evidence that both proteins are correctly folded and functional. Furthermore, we found that both Gpa1 and Gpb1 cross-reacted with specific antibodies to these proteins (Fig. 2C). Consistent with our yeast two-hybrid assays, no interaction between Gpb1 and Gpg1, produced by *in vitro* translation, was detected. This is perhaps not surprising because recent studies have revealed a role for phosducins as molecular chaperones required for Gβγ dimer assembly (Lukov *et al*., 2005).

### Adenylate cyclase interacts with Gpa1 and Gpb1

Although it has been known for some time that G proteins modulate the activity of mammalian AC by binding to the catalytic domain (Tesmer *et al*., 1997), only recently has it been established that *S. pombe* Gpa2 and *S. cerevisiae* Gpa2 bind to the N-terminus of AC to cause its activation in *S. pombe* (Ogihara *et al*., 2004; Ivey and Hoffman, 2005) and *S. cerevisiae* (Peeters *et al*., 2006) respectively.

**Fig. 3:**
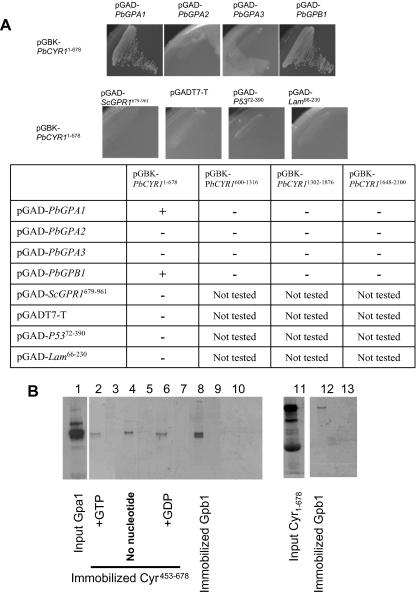
A. Yeast two-hybrid assays indicate that full-length *GPA1* and *GPB1*, but not *GPA2* nor *GPA3*, directly interact with *CYR1*^1−678^. Bait and prey vectors were simultaneously transformed into yeast strain AH109 and plated out on SD/–Leu/–Trp for 3 days. Yeast colonies that grew on SD/–Leu/Trp were restreaked on SD/–Ade/–His/–Leu/–Trp and incubated for a further 3 days. The results growths indicate that pGAD-*PbGPA1* and pGAD-*PbGPB1* could, but pGAD-*PbGPA2* and pGAD-*PbGPA3* could not, directly interact with pGBK-*PbCYR1*^1−678^. In a series of negative controls, pGBK-*PbCYR1*^1−678^ could not interact with pGAD-*ScGPR1*^679−961^, pGADT7-T, pGAD-*P53*^72−390^ and pGAD-*Lam*^66−230^. B. Pull-down assays to demonstrate that Gpa1 and Gpb1 both interact with Gpb1. GST and GST-Cyr^453−678^ were purified from bacteria, loaded onto glutathione sepharose beads before incubation with *in vitro* translated ^35^S-Gpa1 and 10 mM nucleotide. After washing the beads, the proteins were eluted by the addition of 4× NuPAGE LDS sample buffer, followed by boiling at 90°C for 5 min, and separated on a 4–12% NuPAGE gel under denaturing conditions. Bound Gpa1 was detected as a gel band by autoradiography. Lanes 2, 4 and 6 establish that Gpa1 binds to immobilized Cyr1, but there was little difference in apparent affinity after incubation with GTP (lane 2), no nucleotide (lane 4) or GDP (lane 6). Negative controls, using immobilized GST, are shown in lanes 3, 5, 7, 10 and 13. A control using immobilized GST-Gpb1 to pull-down ^35^S-Gpa1, in the presence of 10 mM GDP, shows a more intense band, suggesting that Gpa1-GDP is bound with higher affinity to Gpb1 than to Cyr. Using *in vitro* translated ^35^S-Cyr^1−678^ (lane 11), an interaction with immobilized GST-Gpb1 (lane 12) was detected.

We used two-hybrid analyses to test whether any of the G proteins interact with AC from *P. brasiliensis*. The AC, when analysed with SMART (EMBL), contains four domains: a Ras association and Gα binding domain (RA, positions 1-678) domain (RA, positions); 14 leucine-rich repeats (LRR_TYR domains, positions 752–1244); a serine/threonine phosphatase family 2C catalytic domain (PP2Cc, positions 1341–1627); and an adenylyl/guanylyl cyclase catalytic domain (CYCc domain, positions 1574–1856). The AC cDNA was segmented into four parts with each containing an individual domain and cloned into yeast two-hybrid vectors to make constructions *PbCYR1*^1−678^, *PbCYR1*^600−1316^, *PbCYR1*^1301−1876^ and *PbCYR1*^1347−2100^ that were used to test for interactions with Gpa1–3 and Gpb1 from *P. brasiliensis*. We found that the N-terminus of AC, encoded by the pGBK-*PbCYR1*^1−678^ construct, interacted with Gpa1 and Gpb1, but not with Gpa2 nor Gpa3 (Fig. 3A). Furthermore, a deletion analysis indicated that AC could interact with a pair of WD domains from the extremes of either the N- or C-terminus of Gpb1 (Fig. 2A). We also tested for interactions between the *PbCYR1*^1−678^, *PbCYR1*^600−1316^, *PbCYR1*^1301−1876^ and *PbCYR1*^1347−2100^ constructs, but none were found (data not shown).

To confirm these interactions, we used an N-terminal fragment of Cyr1, comprising residues 453–678, fused to GST, Cyr1_(453−678)_-GST, produced and purified from bacteria, for pull-down assays with *in vitro* translated Gpa1; and an *in vitro* translated N-terminal fragment of Cyr1, comprising residues 1–678, for pull-down assays with the Gpb1–GST fusion protein (Fig. 3B). These assays establishing that both Gpa1 and Gpb1 could interact with the N-terminus of AC and specifically with a region that incorporates the putative Gα and Ras binding domains, between residues 453 and 678 (Fig. 3B). Gpa1 was able to bind Cyr1 in the presence of GTP or GDP or in the absence of nucleotides but, surprisingly, the relative intensities of the bands suggested a preference for Gpa1 in the absence of nucleotides. A similar comparison suggested that Gpa1-GDP had a stronger affinity for Gpb1 than for Cyr1 (Fig. 3B, lane 8). Indeed, in a pull-down assay using immobilized Gpb1-GST with *in vitro* translated, ^35^S-labelled, Cyr1^1−678^ and Gpa1, in the presence of excess Gpa1 and GDP, we did not detect a Cyr1 band, suggesting that Gpa1-GDP binds preferentially to Gpb1 (Fig. 3B, lane 9). However, the strength of these interactions will need to be confirmed by direct measurement when, and if, the proteins can be obtained in sufficient quantities for biophysical studies.

### A transient increase in *CYR1* transcript and cellular cAMP levels correlates with the onset of the morphological switch

**Fig. 4:**
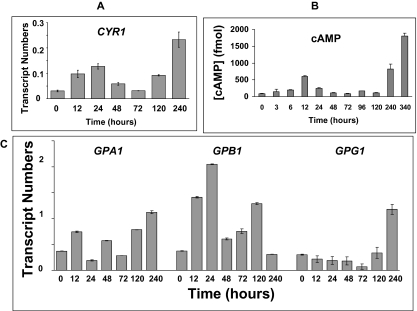
The changes in intracellular cAMP levels correlate with the *CYR1*, *GPA1*, *GPB1* and *GPG1* transcript levels during the mycelium-to-yeast transition. The measured quantity of each *P. brasiliensis* gene mRNA in each of the treated samples was normalized by using the C_T_ values obtained for the *α-tubulin* RNA amplifications run on the same plate. The relative quantification of each *P. brasiliensis* gene and *α-tubulin* gene expression was determined by a standard curve (i.e. C_t_ values plotted against the logarithm of the DNA copy number). The values represent the number of copies of the cDNAs of each *P. brasiliensis* gene divided by the number of copies of the cDNAs of the *α-tubulin* gene. A. A bar chart showing the *CYR1* transcript levels at the indicated times following an increase in temperature from 26°C to 37°C to induce the mycelium-to-yeast transformation. The data represent the average of three independent measurements. B. The corresponding changes in the cellular cAMP levels during the morphological transition from mycelium to yeast are shown in chart B. Intracellular cAMP measurements were made using a non-acetylated EIA procedure (see *Experimental procedures*) and are the average of six assays. C. A bar chart showing the *GPA1*, *GPB1* and *GPG1* transcript levels at the indicated times following an increase in temperature from 26°C to 37°C to induce the mycelium-to-yeast transformation. The data represent the average of three independent measurements.

Real-time reverse transcription polymerase chain reaction (RT-PCR) was used to evaluate the *CYR1* transcript levels, which revealed that it is differentially expressed at higher levels in yeast than in mycelium (Fig. 4A). However, monitoring the transcript levels during the morphological transition revealed a significant transient peak in *CYR1* transcripts after 24 h of the onset of the morphological transition, correlating with the peak in mycelial differentiation, and a further progressive increase in *CYR1* transcripts from about 72 h, as the fungus adopted the yeast form. Considering this behaviour, we sought to determine whether the increase in *CYR1* transcripts correlated with an increase in cellular cAMP levels. We found that the level of cellular cAMP peaked at about 12 h, and then progressively increased from a minimum at 72 h, strongly suggesting that increasing cAMP levels regulate the morphological transition (Fig. 4B). We noted that the *CYR1* transcript levels increased at 24 and 240 h by about 3.2- and 7.5-fold respectively, while the cAMP levels were about 7.5- and 17-fold higher than in mycelium. This behaviour suggests that not only is the expression of AC upregulated, but it is also activated, presumably upon G protein binding.

Our results are intriguing because we previously found that the addition of exogenous dibutyryl-cAMP retarded the morphological transition. Perhaps the cells detect and respond to transient changes in cAMP levels rather than the absolute concentration of cAMP? Consistent with this proposal, we found that adding exogenous dibutyryl-cAMP 12 h after the onset of the morphological transition, when cellular cAMP levels would be maximal, had less effect in retarding the transition compared with its addition at the onset of the transition (i.e. compare charts b and c in Fig. 1C). However, the addition of dibutyryl-cAMP after 120 h, when the cAMP levels had dropped to a minimal level, similar to that in mycelium, induced a partial reversal of the transition (i.e. compare charts d and e in Fig. 1C): at this time, 0.5%, 5.5%, 75.5% and 18.5% of the morphology units were hyphae, differentiating hyphae, transforming yeast and yeast respectively (Fig. 1C, chart d); but after a further 120 h after the addition of the dibutyryl-cAMP, the proportion of these states was 9.3%, 22%, 43% and 25.7% respectively (Fig. 1C, chart e). Presumably because the cells are not synchronized in their development, some are committed to the transition and, accordingly, transform into yeast, but a large proportion of the cells convert back to hyphae. Furthermore, the addition of dibutyryl-cAMP, at concentrations up to 20 mM, did not induce the transformation of yeast at 37°C, indicating that once the cells had passed a certain point in their development, increasing dibutyryl-cAMP was insufficient to reverse this process (data not shown). Indeed, this is consistent with our hypothesis because the cellular cAMP levels are relatively high in yeast, suggesting that the yeast-to-mycelium transition is triggered by decreasing cAMP levels. Consistent with this hypothesis, we could not prevent the temperature-induced yeast-to-mycelium interconversion (e.g. upon decreasing the temperature from 37°C to 26°C) with exogenous dibutyryl-cAMP (data not shown).

### Evidence for a switch from G subunit-signalling during the morphological transition

**Fig. 5:**
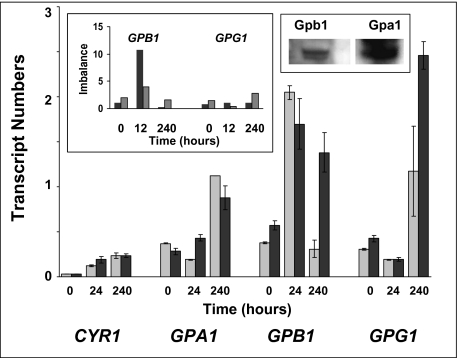
The hindrance of the mycelium-to-yeast transition by dibutyryl-cAMP correlates with an imbalance in Gpa1 and Gpb1 expression. A set of bar charts for the transcript levels of the *CYR1*, *GPA1*, *GPB1* and *GPG1* genes at the indicated times following an increase in temperature from 26°C to 37°C to induce the mycelium-to-yeast transformation in the absence (black bars) and presence (grey bars) of 10 mM dibutyryl-cAMP. The right inset shows the ratio of the *GPB1* and *GPG1* transcripts respectively relative to the number of *GPA1* transcripts in mycelium, and at 12 and 240 h after the onset of the transition to the yeast form. The transcript numbers were determined in the absence (black bars) and presence (grey bars) of 10 mM dibutyryl-cAMP. Data are the average of three independent measurements. The right inset is a Western blot showing that Gpa1 is expressed at a higher level than Gpb1 in yeast.

As our studies indicated that both Gpa1 and Gpb1 could interact with AC, we sought to determine whether there was an imbalance in the concentrations of the G protein subunits during the morphological switch from mycelium to yeast in *P. brasiliensis*. RT-PCR experiments indicated that the *GPA1*, *GPB1* and *GPG1* transcript levels were equivalent in mycelium; but there was a 5.4-fold increase in *GPB1* transcript levels 24 h from the onset of the transition, while those for *GPA1* and *GPG1* declined 2-fold from that in mycelium, so that the *GPB1* transcript levels were more than 10-fold higher than those for *GPA1* and *GPG1*, which were still nearly equivalent (Fig. 5, left inset). Conversely, as the transition approached its end-point, after 240 h, when most cells had adopted the yeast form, the *GPB1* transcript levels dropped to a level 3.7-fold lower than those for *GPA1* and *GPG1.* This behaviour contrasts with that for *RAS* transcript levels that were about 50-fold higher than those for the G protein subunits and fluctuated little during the transition, suggestive of a role in controlling basal cAMP levels (see Fig. S2). We sought to confirm the imbalance in Gpa1 and Gpb1 subunits by Western blotting: comparing the intensities of the bands for the Gpa1 and Gpb1 blots indicated that there was, as predicted, greater expression of Gpa1 than Gpb1 in yeast (Fig. 5, right inset). We did not extend suchanalyses though, because they were complicated by the fact that the Gpa1 antibodies also cross-reacted with higher- and lower-molecular-weight proteins, which could be oligomers and degradation products respectively (data not shown). The presence of the latter would not be surprising if the concentration of Gpa1 needs to be finely controlled in order to modulate cAMP production. Indeed, it would be difficult to comprehend the functional significance of our finding that nucleotide-free Gpa1 and Gpb1 can bind to AC if these were always at equivalent concentrations and preferentially in complex with each other in the absence of GTP. However, we must be cautious in the interpretation of our transcript data because the imbalance in transcript levels may not reflect the difference in protein levels, especially if they are degraded at different rates, and there is a future need to ascertain whether, and how, Gpa1 and/or Gpb1 affect the catalytic activity of AC.

As our morphology studies indicated that the addition of exogenous dibutyryl-cAMP retarded the switch from mycelium to yeast, we sought to determine whether this reflected a change in transcription of AC and/or G proteins. Accordingly, we redetermined the transcript levels in mycelium, 24 h after the onset of the transition, and in yeast, in the presence of 10 mM dibutyryl-cAMP (Fig. 5). There was little change in the *CYR1* transcript levels at any stage in the morphological switch, which could have caused a reduction in cAMP levels to retard the transition. However, after 24 h, there was a notable reduction, from about 10- to 4-fold, in the imbalance in *GPB1* to *GPA1* transcripts. If Gpb1 has a lower affinity than Gpa1 for Cyr1, then this excess of Gpb1 might be insufficient to efficiently curtail the Gpa1 signal by displacing it from Cyr1, potentially retarding the transition. Furthermore, after 240 h there was still a 1.6-fold imbalance in *GPB1* to *GPA1* transcripts; whereas in the absence of exogenous dibutyryl-cAMP, the *GPB1* transcript levels were 3.7-fold lower than *GPA1*. All of the Gpa1 should be complexed in the trimer, and none would be freely available to interact with AC as normal.

## Discussion

Our data are consistent with the morphological transition in *P. brasiliensis* being controlled by changing cAMP levels, with the onset of the transition correlating with a transient increase in cAMP, suggesting that the cAMP-signalling pathway is activated. Furthermore, there is a clear correlation with the changes in cAMP levels and the expression of AC during the transition. However, the fold-increase in *CYR1* transcripts (e.g. 3.2 and 7.5 at 24 and 240 h) was less than that in cAMP levels (e.g. 7.5 and 17 at 24 and 240 h), suggesting that increased cAMP was not simply due to more AC but because the protein is activated. We sought to identify the G proteins most likely responsible for the activation of AC. Most, if not all, filamentous fungi possess three or more Gα proteins, but, to date, only single Gβ and Gγ proteins have been identified. It has remained a mystery as to whether the Gβ and Gγ proteins can form trimeric complexes with the different Gα proteins. In this study, we have established for the first time that the Gβ and Gγ proteins, Gpb1 and Gpg1, interact with only a single Gα protein, Gpa1, in *P. brasiliensis*, presumably to form a Gpa1/Gpb1/Gpg1 trimeric complex. We did not find an interaction between Gpb1 and the other Gα proteins, Gpa2 and Gpa3, which work either independently or perhaps in association with other Gβ mimics (Harashima and Heitman, 2002; Palmer *et al*., 2006; Slessareva *et al*., 2006; reviewed by Hoffman, 2007). We then established that both the Gα and Gβ proteins, Gpa1 and Gpb1, could interact with the N-terminal domain of AC. Previous studies established that, in *S. Pombe*, Gpa2 interacts with the N-terminus of AC to cause its activation (Ogihara *et al*., 2004; Ivey and Hoffman, 2005), while more recently, an interaction between Gpa2 and AC from *S. cerevisiae* was confirmed (Peeters *et al*., 2006). Although there is genetic evidence for the Gβ protein Git5 interacting with and activating AC in *S. pombe* (Landry *et al*., 2000), we have shown a direct interaction between Gβ and a fungal AC that has not previously been demonstrated.

Our studies demonstrate that both Gα and Gβ proteins Gpa1 and Gpb1 bind to a site that lies between residues 453 and 678 of *P. brasiliensis* AC. Similarly, the activity of mammalian ACs is regulated by the binding of Gα and Gβ proteins (Dessauer *et al*., 2002; Diel *et al*., 2006). However, mammalian ACs are integral membrane proteins that share little sequence homology with fungal ACs, which are only associated with the periphery of the membrane. Mammalian ACs have a common topology consisting of two transmembrane domains, M1 and M2, each followed by a cytosolic catalytic domain, C1 and C2 (Krupinski *et al*., 1989). The catalytic activity of mammalian ACs is regulated by the binding of Gαs and Gαi proteins to the C2 and C1 cytoplasmic domains respectively, to increase or decrease the interactions of these domains (Dessauer *et al*., 2002). The effect of binding the Gβγ subunits, apparently to C1, depends upon the AC subtype, for example, activating ACII but inhibiting ACIII (Diel *et al*., 2006). Our data indicate that fungal ACs employ a different mechanism of modulation that involves the binding of G proteins to the N-terminus, but it is not clear how they modulate the activity of the catalytic domain, which is about 1000 residues away. We did not detect any interaction between the RA, LRR_TYR, PP2Cc and CYCc domains of AC, but these might only be induced by the binding of effectors, such as Gpa1 and Gpb1, to the N-terminal domain. The activity of AC in *S. cerevisiae* has been shown to be controlled by its interaction with Sgt1 that binds directly to the LRR domain (Dubacq *et al*., 2002). Similarly, Git7, a homologue of Sgt1, has been shown to control cAMP levels and play a role in glucose-triggered cAMP signalling in *S. pombe* (Schadick *et al.*, 2002). Sequence analyses indicated that Sgt1 has features of a co-chaperone, and it has been proposed to act as a co-chaperone or factor in the assembly or the conformational activation of specific multiprotein complexes (Dubacq *et al*., 2002). Consistent with such a role, recent studies have shown that Sgt1 interacts with Hsp90 (Bansal *et al*., 2004;Lingelbach and Kaplan, 2004). Failure to detect interactions between the different domains of AC in the present investigation might be attributable to the involvement of Sgt1–Hsp90 in stabilizing inter-domain interactions and complex assembly. Considering the growing number of proteins identified as interacting with fungal ACs, this is an attractive hypothesis, as the interactions must occur in a controlled manner.

Our studies indicate that there is an imbalance in Gpa1, Gpb1 and Gpg1 G protein subunits as *P. brasiliensis* undergoes the morphological transition from mycelium to yeast. In mycelium the transcript levels for *GPA1*, *GPB1* and *GPG1* are nearly equivalent, but *GPB1* predominates during the transition, while *GPA1* predominates in yeast. It is notable that the *CYR1*, *GPA1*, *GPB1* and *GPG1* transcripts have extensive leader sequences that incorporate motifs likely to be targeted by RNA binding proteins that can be used to regulate the life times of these transcripts (see *Supplementary material* data). It seems plausible that signal progression is effected by regulating the longevity of these transcripts and of the translated proteins. Presumably, activation of the cAMP-signalling pathway in mycelium will depend upon GTP-induced dissociation of the Gpa1/Gpb1/Gpg1 trimer to release ‘free’ Gpa1 and Gpb1 that can interact with AC. However, remodelling of the cell, as it changes from mycelium to yeast, is a relatively lengthy process, requiring about 10 days for completion. A requirement for GTP to maintain the signal would constitute a metabolic waste over such a time period. An alternative strategy would be to increase the concentration of the protein that activates the signalling process, so that GTP-induced dissociation of the trimeric complex was not subsequently required. Early on in the transition Gpb1 is produced at higher levels than Gpa1 and, as we have established that it only interacts with the Gpa1, and not with Gpa2 or Gpa3, this excess will be ‘free’ to interact directly with AC. However, as we have found that Gpa1 can interact with AC in the absence of GTP, why does not the cell simply produce an excess of Gpa1 as it does in yeast? We note that the excess of Gpa1 in yeast correlates with an increase in the basal cAMP level, suggesting that nucleotide-free Gpa1 can activate AC. Consequently, if Gpa1 was produced in excess during the early stages of the transformation, it would be expected to maintain a high cAMP level, which we have shown, via the addition of dibutyryl-cAMP, actually retards the morphological transition. It seems plausible that Gpb1 serves a role in attenuating the cAMP signal by inactivating AC. Although we have not mapped the precise binding sites for Gpa1 and Gpb1, we have found that both G proteins bind to a domain of AC that incorporates residues 453–678, raising the possibility that they bind to the same site in a competitive manner. Considering the fact that the cAMP levels undergo cyclical changes during the morphological switch, and the effect of exogenous dibutyryl-cAMP in retarding the transition, particularly when cAMP levels are low, it seems likely that these changes in cAMP levels are needed to co-ordinate the activation of sequential steps in the morphological change.

Interestingly, while it is generally believed that it is the Gβγ dimer that is the functional unit, we have found that Gpb1 alone can interact with AC. This behaviour is, however, consistent with the expression of the Gpb1 exceeding that of Gpa1 and Gpg1 during the morphological switch. Gpg1 has a CCAAX box at its C-terminus (e.g. CCMIM) that is the site for prenylation, which is important in targeting the Gβγ dimer to the membrane (Whiteway and Thomas, 1994; Hirschman and Jenness, 1999; Manahan *et al*., 2000). A recent study indicated that there is considerable heterogeneity in the prenylation process, suggesting that this can affect the targeting of the Gβγ dimer, possibly as a means to switch between different signalling pathways (Cook *et al*., 2006). Accordingly, it is worth considering whether the increased expression of Gpb1, above that of Gpg1 and Gpa1, during the morphological transition is not only used to differentially modulate the activity of AC, but could be used to alter the targeting of Gpb1. Recent studies have shown that Gα proteins are segregated into distinct pools that allow specific signalling pathways to be activated at the plasmamembrane and at intracellular membranes (Slessareva *et al*., 2006; Slessareva and Dohlman, 2006).

## Experimental procedures

### Strain and culture

*Paracoccidioides brasiliensis* strain ATCC 90659 was grown as a mycelium form at 26°C and as a yeast form at 37°C in a modified liquid YPD medium (1% yeast extract, 2% neo-peptone and 2% dextrose, pH 6.5) with shaking at 110 r.p.m. for up to 15 days. The primers and plasmids used in this investigation are described in Tables 1 and 2 respectively, and the primers used for gene cloning are shown in Table S1. Exogenous cAMP was added to cultures of *P. brasiliensis* as the non-metabolite cAMP analogue dibutyryl-adenosine 3′-5′-cyclic monophosphoric acid (Sigma).

**Table 1:** Primers used for constructing yeast two-hybrid vectors, quantitative RT-PCR and expression constructs for pull-down assays.

Primer	Sequence (5′→3′; restriction sites underlined)	Description
PbAC-F26(KpnI)	GGTACCAAAATGTCTAGGAGACAGCGGGAGAAAGATAGG	Plasmid construction
PbAC-R34(NotI)	GCGGCCGCGCCGTGCTCGAAGAACTAGAACCAC	Plasmid construction
PbAC-GADF1(NcoI)	GGTACCACCATGGCAAGGAGACAGCGGGAGAAAG	Plasmid construction
PbAC-GADF2(NcoI)	TCGTCCATGGAAGATGAGCTGAATAACTAC	Plasmid construction
PbAC-GADR1(BamHI)	TGATTTGGATCCCGTTGATTCCCAAGTCAGC	Plasmid construction
PbAC-GADR2(BamHI)	CCCCAGGATCCTAAGATGTTTCAAAGAGTTG	Plasmid construction
PbAC-EF1(NdeI)	GGGATTCATATGGTTAATAGCACAGATCTG	Plasmid construction
PbAC-EF2(NdeI)	AGTATCCATATGGGACTGTCTCCATTAACTG	Plasmid construction
PbAC-ER1(XhoI)	CCATGGCTCGAGCGCCGTGCTCGAAGAACT	Plasmid construction
PbAC-ER2(XhoI)	CTCGCGCTCGAGATTGTGATTAAGATAGTC	Plasmid construction
PbGPA1-ExGPAF1	CATATGGGTTGTGGAATGAGC	Plasmid construction
PbGPA1-pYER3	GCCTAGGTCATATCAGTCCACAGAGGCGAAG	Plasmid construction
PbGPA2-F6	CATGGGTTGCGCAAGTTCTCAACCAGTGGA	Plasmid construction
PbGPA2-F7(EcoRI)	CTAGGAATTCATGGGTTGCGCAAGTTCTCA	Plasmid construction
PbGPA2-R6	CTGTTGGCACCTACAGAATCAGGTTGTTGA	Plasmid construction
PbGPA3-F5	TGGTATCAGATAGGATGGGTGGGTGTTGCA	Plasmid construction
PbGPA3-R5	CCCTTTCACAAAATACCAGAATCTTTCAGG	Plasmid construction
PbRAS-F1(NdeI)	CCGCGCTTCATATGCAGCTTTGTCTAAACC	Plasmid construction
PbRAS-R1(EcoRI)	GTGATACTGTAGAATTCCACGAAACCCTC	Plasmid construction
PbGPB-NdeI-F	ACGCTCATATGGCGGCCGATTTGAGCGG	Plasmid construction
PbGPB-BanHI-R	CGATGGATCCCTACCATGCCCAGACCTTGAG	Plasmid construction
PbGPG-NdeI-F	CATATGGCCCCTGCCTACGAGCTTCGAC	Plasmid construction
PbGPG-BamHI-R	GGATCCTTACATGATCATACAGCAGCCACCTGAT	Plasmid construction
pbgpb-NdeI-F	ACGCTCATATGGCGGCCGATTTGAGCGG	pAD-WD1
gpbWD-1R	GCTAGGATCCTAATCGGAGATGATTAGTTTCC	pAD-WD1
pbgpb-NdeI-F	ACGCTCATATGGCGGCCGATTTGAGCGG	pAD-WD2
gpbWD-2R	GCTAGGATCCTAATTATAGATGGAACAGATG	pAD-WD2
pbgpb-NdeI-F	ACGCTCATATGGCGGCCGATTTGAGCGG	pAD-WD3
gpbWD-3R	GCTAGGATCCTAATCCCATAGCATACAGGTC	pAD-WD3
pbgpb-NdeI-F	ACGCTCATATGGCGGCCGATTTGAGCGG	pAD-WD4
gpbWD-4R	GCTAGGATCCTAATCCCAGAGTTTAGCAAAGG	pAD-WD4
pbgpb-NdeI-F	ACGCTCATATGGCGGCCGATTTGAGCGG	pAD-WD5
gpbWD-5R	CGTAGGATCCTAATCGAATAGACGGCAGGTGG	pAD-WD5
pbgpb-NdeI-F	ACGCTCATATGGCGGCCGATTTGAGCGG	pAD-WD6
gpbWD-6R	GCTAGGATCCTAGTCCCAGACCTTGCACTCAT	pAD-WD6
pbgpb-NdeI-F	ACGCTCATATGGCGGCCGATTTGAGCGG	pAD-WD13
gpbWD-1R	GCTAGGATCCTAATCGGAGATGATTAGTTTCC	pAD-WD13
Gpb-WD3-F	GGATCCCTTTCCTCTCGAGAAGGTCC	pAD-WD13
gpbWD-3R	GCTAGGATCCTAATCCCATAGCATACAGGTC	pAD-WD13
Gpb-WD27-R	CATATGGCATACACAACAAACAAAGTGCAC	pAD-WD27
pbgpb-BanHI-R	CGATGGATCCCTACCATGCCCAGACCTTGAG	pAD-WD27
gpbWD-67-F	CATATG ATCCGCGCGGATAGAGAACTTAATAC	pAD-WD67
pbgpb-BanHI-R	CGATGGATCCCTACCATGCCCAGACCTTGAG	pAD-WD67
ScGPR1-F1	CGGGATCCGAAGTGTGACGAATAAAGC	Plasmid construction
ScGPR1-F3	CGGGATCCATATGATAACTGAGGGATTTCCCCCG	Plasmid construction
ScGPR1-F4	CCGGATCCGTGAAAGTAAAAGAATTAAAGCGC	Plasmid construction
ScGPR1-F5	CCGGATCCAGGAAAAACCTTGGAACTATTCATG	Plasmid construction
ScGPR1-R1	CGGGATCCATTTTCAAACATCGCGATAC	Plasmid construction
ScGPR1-R3	CCGGATCCTTAGATTCTTTTGAATTTGTGCC	Plasmid construction
ScGPA2-F1	CCGGATCCTGGGTCTCTGCGCATCTTCA	Plasmid construction
ScGPA2-R1	CCGGATCCGCTGTGCATTCATTGTAACAC	Plasmid construction
3′BD screening	TGGCTGCAAGCGCGCAAAAAACCCCTCAAGAC	Plasmid construction
5′BD screening	TCATCGGAAGAGAGTAGTAACAAAGGTCAAAGA	Plasmid construction
pGADT-linker-gamma-F1	CCGCTCCGGCCCTGACCCCGGCGGATGTGGCCCGCAGCAT GGCCCCTGCCTACGAGCTTCGACCCG	For construct of PbGpb-linker-PbGpgamma fusion gene
pGADT-linker-gamma-F2	GGATCCCGTATTAAAAACGGCAGCGGTGCGGCAGCCCCGAAA GCCGCTCCGGCCCTGACCCCGGCGGATG	For construct of PbGpb-linker-PbGpgamma fusion gene
pGADT-Gpb-R2(TAG)	GGATCCCCATGCCCAGACCTTGAGCAGAGAATC	For construct of PbGpb-linker-PbGpgamma fusion gene
Real-time PCR		
Alpha P-alpha	CCAGAACCAGGCAGTCCAAA FAM-CACCTGCCTAACAAGATTGGACCAGG5G	Real-time PCR of α-tubulin
P-gpa1_Pb p-gpa1_Pb	GTACCGCCACCTACGTCGAACATACGG5AC-FAM ATGTCCTTCGCTCCCGTGTTA	Real-time PCR of *gpa1*
P-Gpb_Pb p-Gpb_Pb	CACGGCGAGAAGGTCCAACCCG5G-FAM TCGCCGGAAGAAGTGATGATT	Real-time PCR of *gpb1*
P-Gpg_Pb p-Gpg_Pb	CGGTTGTCAATCTGTCCCCAAAC5G-FAM CGGCCATGTCACTCATCAACT	Real-time PCR of *gpg1*
P-PbAden_cyc p-PbAden_cyc	CACATTCAAAGTTTCGTGGGAAGGAATG5G-FAM AGAGGCCGATTCTCATGCAA	Real-time PCR of *cyr1*
Protein overexpression		
pGEX-6P-3 Cyr1453–678_Pb (BamHI and NotI)	AAGGATGGATCCGATAAAACCCATCAGGATAACTTTG CATATCGCGGCCGCTTAGTGGCTAAACTTTTGGTTCTCGTG	Plasmid construction
pGEX-6P-3 Gpb1 (BamHI and NotI)	ATACATGGATCCATGGCGGCCGATTTGAGCGGCG ATATCTGCGGCCGCCTACCATGCCCAGACCTTG	Plasmid construction

In most cases, the same primers were used to make constructs in both pGADT7 and pGBKT7 for yeast two-hybrid screens.

**Table 2:** Plasmid used in this study.

Plasmid name	Vector	Insert and cloning description
pGEMTE-cPbAC-F26R34-1	pGEM T Easy (Promega)	The insert is the full-length *PbCYR1* cDNA amplified with PbAC-F26 and PbAC-R34
pGBK-PbCYR1_(1−678)_	pGBKT7 (Clontech)	*PbCYR1* cDNA fragment (NcoI/BamHI) obtained by PCR with PbAC-GADF1 and PbAC-GADR1 cloned into pGBKT7 (NcoI/BamHI)
pGBK-PbCYR1_(600−1316)_	pGBKT7 (Clontech)	*PbCYR1* cDNA fragment (NcoI/BamHI) obtained by PCR with PbAC-GADF2 and PbAC-GADR2 cloned into pGBKT7 (NcoI/BamHI)
pGBK-PbCYR1_(1302−1876)_	pGBKT7 (Clontech)	*PbCYR1* cDNA fragment (NdeI/XhoI) obtained by PCR with PbAC-EF1 and PbAC-ER2 cloned into pGBKT7 (NdeI/SalI)
pGBK-PbCYR1_(1648−2100)_	pGBKT7 (Clontech)	*PbCYR1* cDNA fragment (NdeI/XhoI) obtained by PCR with PbAC-EF2 and PbAC-ER1 cloned into pGBKT7 (NdeI/SalI)
pGAD-PbCYR1_(1−678)_	pGADT7 (Clontech)	*PbCYR1*^1−678^ fragment from pGBK-PbCYR1_(1−678)_ (NdeI/BamHI) cloned into pGADT7 (NdeI/BamHI)
pGAD-PbCYR1_(600−1316)_	pGADT7 (Clontech)	*PbCYR1*^600−1316^ fragment from pGBK-PbCYR1_(600−1316)_ (NdeI/BamHI) cloned into pGADT7 (NdeI/BamHI)
pGAD-PbCYR1_(1302−1876)_	pGADT7 (Clontech)	*PbCYR1* cDNA fragment (NdeI/XhoI) obtained by PCR with PbAC-EF1 and PbAC-ER2 cloned into pGADT7 (NdeI/XhoI)
pGAD-PbCYR1_(1648−2100)_	pGADT7 (Clontech)	*PbCYR1* cDNA fragment (NdeI/XhoI) obtained by PCR with PbAC-EF2 and PbAC-ER1 cloned into pGBKT7 (NdeI/XhoI)
pGEMTE-cPbGPA1-ExGPAF1pYER3-9(T7>SP6)	pGEM T Easy (Promega)	RT-PCR product with PbGPA1-ExGPAF1 and PbGPA1-pYER3 from *Pb* mRNA cloned into pGEM T Easy. The insert is the full-length *PbGPA1* cDNA
pGEMTE-cPbGPA2-F6R6-2(SP6>T7)	pGEM T Easy (Promega)	RT-PCR product with PbGPA2-F6 and PbGPA2-R6 from *Pb* mRNA cloned into pGEM T Easy. The insert is the full-length *PbGPA2* cDNA
pGEMTE-cPbGPA3-F5R5-2(T7>SP6)	pGEM T Easy (Promega)	RT-PCR product with PbGPA3-F5 and PbGPA3-R5 from *Pb* mRNA cloned into pGEM T Easy. The insert is the full-length *PbGPA3* cDNA
pGBK-PbGPA1	pGBKT7 (Clontech)	Insert (NdeI) from pGEMTE-cPbGPA1-ExGPAF1pYER3-9 cloned into pGBKT7 (NdeI)
pGAD-PbGPA1	pGADT7 (Clontech)	*PbGPA1* insert (NdeI digested) from pGBK-PbGPA1 cloned into pGADT7 (NdeI)
pGBK-PbGPA2	pGBKT7 (Clontech)	PCR product (EcoRI digested) with PbGPA2-F7 and T7 as primers and pGEMTE-cPbGPA2-F6R6-2 as template, cloned into pGBKT7 (EcoRI digested)
pGAD-PbGPA2	pGADT7 (Clontech)	*PbGPA2* insert (NdeI/SalI digested) from pGBK-PbGPA2 cloned into pGADT7 (NdeI/XhoI)
pGBK-PbGPA3	pGBKT7 (Clontech)	PCR product (EcoRI digested) with PbGPA3-F8 and SP6 as primers and pGEMTE-cPbGPA3-F5R5-2 as template, cloned into pGBKT7 (EcoRI digested)
pGAD-PbGPA3	pGADT7 (Clontech)	*PbGPA3* insert (NdeI/BamHI digested) from pGBK-PbGPA3 cloned into pGADT7 (NdeI/BamHI)
pGBK-PbGPB1	pGBKT7 (Clontech)	*PbGPB1* cDNA (NdeI/BamHI) cloned into pGBKT7 (NdeI/BamHI)
pGAD-PbGPB1	pGADT7 (Clontech)	*PbGPB1* cDNA (NdeI/BamHI) cloned into pGADT7 (NdeI/BamHI)
pGBK-PbRAS	pGBKT7 (Clontech)	*PbRAS* cDNA (NdeI/EcoRI) cloned into pGBKT7 (NdeI/EcoRII)
pGAD-PbRAS	pGADT7 (Clontech)	*PbRAS* insert (NdeI/EcoRI digested) from pGBK-PbRAS cloned into pGADT7 (NdeI/EcoRI)
pGBKT7-53	pGBKT7 (Clontech)	From Clontech
pGAD-P53_(72−390)_	pGADT7 (Clontech)	*P53* insert (NdeI/BamHI digested) from pGBKT7-P53, cloned into pGADT7 (NdeI/BamHI)
pGBKT7-Lam	pGBKT7 (Clontech)	From Clontech
pGAD-Lam_(66−230)_	pGADT7 (Clontech)	*Lam* insert (NdeI/BamHI) from pGBKT7-Lam cloned into pGADT7 (NdeI/BamHI)
pGADT7-T	pGADT7 (Clontech)	From Clontech
pGEMTE-ScGPR1-F1R1-17	pGEM T Easy (Promega)	The insert is the full-length *ScGPR1* amplified with Sc-GPR1-F1 and ScGPR1-R1
pGAD-ScGPR1_(679−961)_-F5R1	pGADT7 (Clontech)	PCR product (BamHI digested) with ScGPR1-F5 and ScGPR1-R1 as primers, and pGEMTE-ScGPR1-F1R1-17 as template, cloned into pGADT7 (BamHI digested); corresponding to C-terminal cytoplasmic domain
pGAD-ScGPR1_(274−621)_-F4R3	pGADT7 (Clontech)	PCR product (BamHI digested) with ScGPR1-F4 and ScGPR1-R3 as primers, and pGEMTE-ScGPR1-F1R1-17 as template, cloned into pGADT7 (BamHI digested); corresponding to the third cytoplasmic loop
pGAD-ScGPR1_(1−961)_-F3R1	pGADT7 (Clontech)	PCR product (BamHI digested) with ScGPR1-F3 and ScGPR1-R1 as primers, and pGEMTE-ScGPR1-F1R1-17 as template, cloned into pGADT7 (BamHI digested); corresponding to full-length *ScGPR1*
pGBK-ScGPR1_(1−961)_-F3R1	pGBKT7 (Clontech)	Insert (BamHI digested) from pGAD-ScGPR1_(1−961)_-F3R1 cloned into pGBKT7
pGBK-ScGPA2-F1R1	pGBKT7 (Clontech)	PCR product (BamHI digested) with ScGPA2-F1 and ScGPA2-R1 as primers, and Sc DNA as template, cloned into pGBKT7 (BamHI digested); corresponding to full-length ScGPA2
pAD-PbGPB1	pGADT7 (Clontech)	1062 bp *PbGPB1* gene linked into pGADT-7 at the sites of NdeI and BamHI
pBD-PbGPB1	pGBKT7 (Clontech)	1062 bp *PbGPB1* gene linked into pGBKT-7 at the sites of NdeI and BamHI
pAD-PbGPB1-TAG	pGADT7 (Clontech)	1059 bp *PbGPB1* gene without stop code was linked into pGADT-7 at the site of NdeI and BamHI
pBD-PbGPB1-TAG	pGBKT7 (Clontech)	1059 bp *PbGPB1* gene without stop code was linked into pGBDT-7 at the site of NdeI and BamHI
pAD-PbGPG1	pGADT7 (Clontech)	276 bp *PbGPG1* gene linked into pGADT-7 at the sites of NdeI and BamHI
pBD-PbGPG1	pGBKT7 (Clontech)	276 bp PbGpg gene linked into pGBKT-7 at the sites of NdeI and BamHI
pAD-PbGPB1-link-PbGPG1	pGADT7 (Clontech)	PbGpb-flexible linker-PbGpg was ligated into pGADT-7 at the site of NdeI and BamHI
pBD-PbGPB1-link-PbGPG1	pGBKT7 (Clontech)	PbGpb-flexible linker-PbGpb was ligated into pGBKT-7 at the site of NdeI and BamHI
pAD-WD1	pGBKT7 (Clontech)	The first WD domain of PbGpb1 with a stop codon linked into pGADT-7 at the sites of NdeI and BamHI
pAD-WD2	pGADT7 (Clontech)	The region encoding from the N-termini to the end of the second WD domain of PbGpb1 with a stop codon linked into pGADT-7 vector at the sites of NdeI and BamHI
pAD-WD3	pGADT7 (Clontech)	The region encoding from the N-termini to the end of the third WD domain of PbGpb1 with a stop codon linked into pGADT-7 vector at the sites of NdeI and BamHI
pAD-WD4	pGADT7 (Clontech)	The region encoding from the N-termini to the end of the fourth WD domain of PbGpb1 with a stop codon linked into pGADT-7 vector at the sites of NdeI and BamHI
pAD-WD5	pGADT7 (Clontech)	The region encoding from the N-termini to the end of the fifth WD domain of PbGpb1 with a stop codon linked into pGADT-7 vector at the sites of NdeI and BamHI
pAD-WD6	pGADT7 (Clontech)	The region encoding from the N-termini to the end of the sixth WD domain of PbGpb1 with a stop codon linked into pGADT-7 vector at the sites of NdeI and BamHI
pAD-WD13	pGADT7 (Clontech)	The region encoding a fragment containing the first and third WD domains of PbGpb1 with a start codon linked into pGADT-7 vector at the sites of NdeI and BamHI
pAD-WD27	pGADT7 (Clontech)	The region encoding a fragment from the second WD domain to the end of PgGpb1 with a start codon linked into pGADT-7 vector at the sites of NdeI and BamHI
pAD-WD67	pGADT7 (Clontech)	The region encoding a fragment from the sixth WD domain to the end of PgGpb1 with a start codon linked into pGADT-7 vector at the sites of NdeI and BamHI
pAD-WD17	pGADT7 (Clontech)	Deletion of a SalI fragment from the region encoding the fragment between the first and seventh WD domains of PbGpb1 in pAD-PbGPB1
pGEX-6P-3 Cyr1_(453−678)_	pGEX-6P-3 (GE Healthcare)	The region encoding the cDNA fragment of *CYR1*, 225 bp (Gα binding domain and Ras association domain) linked to pGEX-6P-3 vector (GST fusion) at the sites of BamHI and NotI
pGEX-6P-3 Gpb1	pGEX-6P-3 (GE Healthcare)	1062 bp *PbGPB1* gene linked into pGEX-6P-3 (GST fusion) vector at the sites of BamHI and NotI

### Microscopy

For the microscopic assays, the different morphotypes were transferred to fixative solution (3.7% formaldehyde, 50 mM sodium phosphate buffer pH 7.0, 0.2% Triton X-100) for 120 min at room temperature. Then, they were briefly rinsed with PBS buffer (140 mM NaCl, 2 mM KCl, 10 mM NaHPO_4_, 1.8 mM KH_2_PO_4_, pH 7.4) and mounted on the slides. The material was photographed using a Zeiss epifluorescence microscope.

### RNA extraction

For the real-time RT-PCR experiments, yeast cells and mycelium were disrupted with glass beads and grinding in liquid nitrogen respectively, and immediately mixed with Trizol (Gibco-BRL) for RNA extraction following the supplier's recommendations. To verify the RNA integrity, 20 μg of RNA from each treatment was fractionated in 2.2 M formaldehyde, 1.2% agarose gel, stained with ethidium bromide, and visualized with UV light. The presence of intact 28S and 18S ribosomal RNA bands was used as a criterion to verify whether the RNA was intact. RNase-free DNase treatment was performed in a final volume of 100 μl containing 40 mM Tris-HCl pH 7.5 and 6 mM MgCl_2_, 1 μl of RNasin (40 U μl^−1^, Promega, USA), 10 μl of RNase-free DNase (1 U μl^−1^, Promega or Life Technologies, USA), 2.5 μl of 200 mM DTT, and 10 μg of total RNA. The reaction was incubated at 37°C for 60 min and stopped by incubating at 70°C for 30 min. The absence of DNA contamination after the RNase-free DNase treatment was verified by PCR amplification of the *GP43* gene.

### Construction of cDNA libraries

A *P. brasiliensis* yeast cDNA library was constructed in the vector pDNR-LIB using the Creator SMART cDNA Library (Clontech) according to the manufacturer's instructions. The *CYR1*, *GPA1*, *GPA2*, *GPA3*, *GPB1*, *GPG1* and *RAS* genes were cloned from the Creator pDNR Library as described in *Supplementary methods*.

### Real-time PCR and RT-PCR reactions

All the real-time PCR and RT-PCR reactions were performed using an ABI Prism 7700 Sequence Detection System (Perkin-Elmer Applied Biosystems, USA). Taq-ManR EZ RT-PCR kits (Applied Biosystems, USA) were used for RT-PCR reactions. The thermocycling conditions comprised an initial step at 50°C for 2 min, followed by 30 min at 60°C for reverse transcription, 95°C for 5 min, and 40 cycles at 94°C for 20 s and 60°C for 1 min. Taq ManR PCR reagent kits were used for PCR reactions. As there is no ideal control for gene expression, we first compared several genes as normalizers for the expression experiments, such as those encoding α-tubulin, hexokinase and a translation factor. We have seen no difference by using any of these normalizers. Accordingly, the calibrator gene used for the expression experiments was the *α-tubulin* gene (data not shown). The reactions and calculations were performed according to Semighini *et al*. (2002). Primer and probe sequences are described in Table 1.

### Subcloning of genes for yeast two-hybrid analysis

The *GPA1, GPA2, GPA3, GPB1* and *GPG1* genes, and fragments of the *CYR1* and *GPB1* gene, were subcloned into the vectors pGBKT7 and pGADT7 for use in yeast two-hybrid screens (Table 2).

### Construction of random mutagenesis libraries for yeast two-hybrid screening

Random mutagenesis libraries for *GPA1*, *GPA2*, *GPA3* and *GPB1* from *P. brasiliensis*, *GPR1* from *S. cerevisiae*, and mammalian *P53* and *LAM* were constructed using the GeneMorph II Random Mutagenesis kit (Stratagene) and cloned into the prey vector pGADT7 to create the libraries pGAD-PbGPA1-RM-Lib, pGAD-PbGPA2-RM-Lib, pGAD-PbGPA3-RM-Lib, pGAD-PbGPB-RM-Lib, pGAD-ScGPR1-F5R1-RM-Lib, pGAD-P53-RM-Lib and pGAD-Lam-RM-Lib respectively for yeast two-hybrid screening.

### Yeast two-hybrid analysis and screening

The Matchmaker Two-Hybrid System 3 (Clontech) was used to test for protein–protein interactions and to screen librariesfor potential interacting protein partners. For the former purpose, transformants were generated by introducing both bait and prey vectors into yeast strain AH109 simultaneously. For the latter, bait vectors were introduced into AH109 first, followed by sequential transformation with 20 μg of prey library plasmids. Experimental procedures were conducted in accord with the Matchmaker GAL4 Two-Hybrid System 3 manual and the Yeast Protocol Handbook (Clontech). Protein interactions were identified by observing the growth of transformants on SD-Ade/–His/–Leu/–Trp plates. After screening random mutagenesis libraries, candidate transformants were twice restreaked on SD-Ade/–His/–Leu/–Trp plates to allow loss of non-interacting prey vectors. Interacting prey vectors were purified using a Yeast Plasmid Isolation kit (Clontech) and subjected to DNA sequence analysis to confirm the identities of the interacting gene products.

### Assays for cAMP production

*Paracoccidioides brasiliensis* mycelium growing in modified liquid YPD media at 26°C was subjected to an increase in temperature to 37°C to induce the morphological transition to the yeast form. Cells were harvested at different times during the transition and immediately stored at −80°C. To thawed cells, collected by centrifugation, 4% formic acid was added and agitated for 5 h to disrupt the cells. The cell debris was removed by centrifugation and the supernatant was lyophilized. Subsequently, the lyophilized pellet was made up in assay buffer, containing 2.5% dodecyl-trimethyl-ammonium-bromide, and was assayed using a Biotrak Enzyme Immuno Assay (EIA) kit from Amersham, according to the manufacturer's protocol 3. Measurements were normalized by using an equivalent wet weight of cells during the disruption procedure.

### Expression of GST-Gpb1 and GST-Cyr1^453−678^

*GPB1* and *CYR1*^*453−5678*^ were cloned into pGEX6p-3, to enable expression of GST fusion proteins, and transformed into *Escherichia coli* codon plus cells, which were grown in 2YT, at 25°C with shaking at 200 r.p.m., before induction with 0.1 mM IPTG. Cells were harvested by centrifugation, resuspended and disrupted by passage through a Constant Systems cell disrupter; 0.1% Triton X-100 was added to the disrupted cells and the debris collected by ultracentrifugation. The supernatant was mixed with GST beads and incubated on a rotator for 30 min at 4°C, loaded into a glass column, and washed with PBS and finally with GST elution buffer (50 mM Tris/HCl pH 8.0, 10 mM glutothione). The elution fractions were run on 4–12% SDS-PAGE (NuPAGE precast) polyacrylamide gels. The protein concentrations were measured using a BCA^TM^ protein assay kit (Pierce). These procedures typically yielded 7 mg ml^−1^ GST-Cyr1^453−678^, 5 mg ml^−1^ GST-Gpb and 3 mg ml^−1^ GST.

### *In vitro* translation of Gpa1, Gpg1 and Cyr1^1−678^

Gpa1, Gpg1 and Cyr1^1−678^ were synthesized by an *in vitro* coupled transcription and translation system (Promega), labelling the proteins with Redivue^TM^l-^35^S-methionine (Amersham), using rabbit reticulocyte lysate. This was necessary because our attempts to overexpress these proteins, as well as full-length Cyr1, either as His-tagged or as GST-tagged proteins, always resulted in the production of inclusion bodies. The yeast two-hybrid pGBKT7 vectors, into which each gene or gene fragment had been cloned, were used as the template for the *in vitro* translation. The reaction was incubated at 30°C for 2 h, and 2.5 μl of the translated samples was loaded onto a gel to verify the translation. The translated proteins were stored at 4°C.

### Pull-down assays

GST pull-down assays were performed with GST fusion proteins as bait and proteins labelled with ^35^S, produced by TNT-coupled transcription/translation (Promega), as prey. The GST proteins were immobilized on 40 μl glutathione sepharose 4B beads (GE Healthcare), which had been preblocked with 200 μl of binding buffer (20 mM HEPES pH 7.9, 600 mM NaCl, 0.1% Tween 20, 5% glycerol, 1 mM DTT, 5% milk and 1% BSA) for 10–15 min at room temperature with 10 μl of EDTA-free protease inhibitor (1/4 tablet in 0.2 mlPBS) (Roche); to which was added 10 μl of *in vitro* translated protein and, in some assays, 10 mM GTP or GDP, and incubated on an end-over rotator at room temperature for 2 h. To facilitate comparisons between different pull-down experiments, we always utilized the same batch of *in vitro* translated product at an equivalent concentration. The beads were washed seven times with buffer (20 mM HEPES pH 7.9, 600 mM NaCl, 0.1% Tween 20, 5% glycerol, 1 mM DTT), and then proteins were eluted by the addition of 4× NuPAGE LDS sample buffer (Invitrogen), followed by boiling at 90°C for 5 min. The proteins were separated on 4–12% NuPAGE (precast Bis-Tris) gels. The gels were fixed with 20% ethanol and 10% acetic acid for 30 min, and then soaked in 5–10 ml of fluorographic reagent NAMP 100 (Amersham Biosciences) to amplify the signal. The gels were dried at 80°C for 35 min under vacuum and autoradiographed (2–3 days exposed at −80°C). Each assay was repeated three times with a different batch of *in vitro* translated product to confirm the results.

### Western blots

Antibodies were to the GST tag (Novagen) or commercially produced polyclonal antibodies (Invitrogen) raised in rabbits to specific oligopeptides: Gpa1 – CFR RSR EYQ LND SAR and Gpb1 – CDI RAD REL NTY QSD.

Proteins were separated by SDS-PAGE (on 4–12% polyacrylamide gels) and electrotransferred to PVDF membranes. Blots were incubated with the respective antibodies (e.g. anti-GST at 1:12 500 dilution and specific antibodies at 1:2500 dilution). Alkaline-phosphatase-conjugated anti-mouse IgG (1:2500 dilution) and horseradish peroxidase-conjugated anti-rabbit IgG (1:2500 dilution) were used as secondary antibodies for GST and specific protein blots respectively.

### Nucleotide sequence accession number

The GenBank accession numbers for the *P. brasiliensis* genes used in this study are: *CYR1* (AAS01025), *GPA1* (AAT40562), *GPA2* (AAT40564), *GPA3* (AAT40563), *GPB1* (AAT40565), *GPG1* (EF687895) and *RAS* (AY547438).

## Supplementary material

The following supplementary material is available for this article:

Fig. S1 The phylogenetic relationship between *Paracoccidioides brasiliensis* and other fungal cAMP-signalling proteins – a neighbour-joining bootstrap tree is derived from the amino-acid sequence alignments of fungal (A) adenylate cyclase (AC) (B) Gb, (C) Gg and (D) Ga proteins, using the VectorNTi 6.0 align program (Informax).

Fig. S2 The RAS transcript levels during the mycelium-toyeast transition.

Table S1 Primers used for gene cloning.

This material is available as part of the online article from: http://www.blackwell-synergy.com/doi/abs/10.1111/j.1365-2958.2007.05824.x

(This link will take you to the article abstract).
