# Supplementary material for: The cAMP pathway is important for controlling the morphological switch to the pathogenic yeast form of Paracoccidioides brasiliensis
Source: Mol Microbiol. 2007 Aug 1;65(3):761–79. doi: 10.1111/j.1365-2958.2007.05824.x (PMC2064555; doi:10.1111/j.1365-2958.2007.05824.x)

Supplementary Information

Supplementary Methods

RNA Extraction – To provide RNA for use in constructing cDNA libraries, the yeast cell pellet was re-suspended in fresh YPD media and the cells grown, with shaking at 160 rpm, into log phase for 6 hours at 37ºC. Subsequently, the cells were precipitated by centrifugation and re-suspended in RNAlater (Ambion) and stored in -80°C. On the day of RNA extraction, RNAlater was discarded by centrifugation and the cells were disrupted by maceration after freezing in liquid nitrogen. Total RNA was extracted by using RNeasy Midi Kit (Qiagen) according to manufacturer's instruction; and from this, mRNA was extracted using an Oligotex Midi Kit (Qiagen).

Cloning the *CYR1* gene that encodes adenylate cyclase - Generally, the strategy for cloning *PbCYR1* included library screening and PCR amplifications walking on both genomic DNA and cDNA. Initially, two degenerate primers, PbAC-F1 and PbAC-R1, were designed corresponding to conserved amino acid residues VFTDIKNST and MDY(YCF)GPMVN, respectively (Binz *et al*., 1998). A single 389 bp fragment, gPbAC-F1R1 was amplified from *P. brasiliensis* genomic DNA. This fragment was then labelled with fluorescein using Gene Images Random Prime Labelling Kit (Amersham) and used for screening the λZAPII-cDNA library. Two clones were identified, with one named PbAC-2, having a 4.0 kb insert. Sequencing of PbAC-2 showed high homology to other fungal adenylate cyclases but it was a stretch of genomic DNA, indicating the existence of residual genomic DNA in the mRNA preparation used for construction of the λZAPII-cDNA library. On the basis of the PbAC-2 sequence, two specific primers, PbAC-F8 and PbAC-R30, were designed for downstream and upstream RACE PCR. By using these 2 specific primers and 2 anchor primers (M13R1 and M13F1), 2 fragments, named cPbAC-R30M13F1 and cPbAC-F8M13R1-B6, were amplified from the pDNR-cDNA library plasmids. These 2 fragments turned out to be cDNA as indicated by the existence of an intron-splicing and a polyA tail in cPbAC-F8M13R1-B6. Another cDNA fragment, cPbAC-F15R19, connecting cPbAC-R30M13F1 and cPbAC-F8M13R1-B6, was subsequently amplified. An upstream cDNA fragment, cPbAC-R7SIV1, was amplified by RACE

PCR after RT reaction with PbAC-R7. In the same manner, 2 additional fragments toward the 5' end of the cDNA were amplified, namely cPbAC-R12SIV1 and cPbAC-R21SIV2-12, with the start codon ATG in the later. Genomic DNA amplification was based on the sequence of the finished cDNA sequence. The genomic *PbAC1* sequence was finished by amplifying a sequence, gPbAC-F12R30, from genomic DNA and another fragment, gPbAC-R16AP21, from a Genomic Walker library in λDASH. A full length cDNA construct for the *CYR1* gene was made by cloning cDNA fragments from the *P. brasiliensis* cDNA pDNR library into pBSK (Invitrogen) and PGEM T Easy (Promega). This was used as the PCR target to generate fragments of the *CYR1* gene that encoded Cyr1$^{(1-678)}$, Cyr1$^{(600-1316)}$, Cyr1$^{(1301-1876)}$ and Cyr1$^{(1347-2100)}$.

Cloning of the 3 iso-forms of the Gα protein genes *gpa1, 2 and 3* - The strategy for cloning of *PbGPA1-3* was PCR amplifications walking on both genomic DNA and cDNA. Initially, two degenerate primers, PbGPA-DGF5 and PbGPA-DGR2, were designed corresponding to conserved amino acid residues (AS)GESGKST and RKKWIHC, respectively. Three fragments of 578 bp, 680 bp and 644 bp were amplified from genomic DNA, and designated *PbGPA1*, *PbGPA2* and *PbGPA3* accordingly. These fragments were sequenced and specific primers were designed, with which, fragments upstream and downstream of the genes were amplified from both a Genomic Walker library and the pDNR-cDNA library.

On the basis of gPbGPA-DGF5R2 (578 bp, *PbGPA1*) sequence, two specific primers, PbGPA1-F1 and PbGPA1-R2, were designed for upstream and downstream SSP-PCR. By using these 2 specific primers and 2 anchor primers (M13R1 and M13F1), 2 cDNA fragments, namely cPbGPA1-F1M13R1 and cPbGPA1-R2M13F1, were amplified from the pDNR-cDNA library plasmids. An upstream genomic DNA fragment, gPbGPA1-R4AP21-3, was amplified by SSP-PCR from the Genome Walker library. A downstream genomic DNA fragment, gPbGPA1-F2AP3-2, was amplified as well. The genomic DNA sequence was finished by amplifying a fragment, gPbGPA1-F7R1, bridging gPbGPA1-R4AP21-3 and gPbGPA1-F2AP3-2. The full-length *PbGPA1* cDNA

sequence was amplified with 2 specific primers, PbGPA1-ExGPAF1 and PbGPA1-pYER3, which included the ATG start codon and TGA stop codon respectively.

On the basis of gPbGPA-DGF5R2 (680 bp, *PbGPA2*) sequence, 4 specific primers, PbGPA2-F1, PbGPA2-F2, PbGPA2-R1 and PbGPA2-R2 were designed for cloning of *PbGPA2* by SSP-PCR. By using specific primers PbGPA2-F1 and PbGPA2-R1, and an anchor primer AP3, 2 genomic DNA fragments, namely gPbGPA2-F1AP3 and gPbGPA2-R1AP3, were amplified from the Genome Walker library. Another two cDNA fragments were amplified, namely cPbGPA2-R2M13F1 and cPbGPA2-F2M13R1, from the pDNR-cDNA library. Then a genomic DNA fragment was amplified using specific primers PbGPA2-R2 and PbGPA2-F4 (designed on the basis of the sequence of cPbGPA2-R2M13F1) from *P. brasiliensis* genomic DNA. A further upstream genomic DNA sequence, gPbGPA2-R8AP4-4, was amplified from the Genome Walker library. The full-length cDNA of *PbGPA2*, namely cPbGPA2-F6R6, was amplified from *P. brasiliensis* mRNA with PbGPA2-F6 and PbGPA2-R6, which included the start and stop codons, respectively.

On the basis of gPbGPA-DGF5R2 (644 bp, *PbGPA3*) sequence, two specific primers PbGPA3-F1 and PbGPA3-R1 were designed for cloning *PbGPA3* by SSP-PCR. By using the 2 specific primers and an anchor primer AP3, 2 genomic DNA fragments, gPbGPA3-F1AP3-2 and gPbGPA3-R1AP3-2, were amplified from the Genome Walker library. A new primer PbGPA3-R4 was designed, and a further upstream genomic fragment, gPbGPA3-R4AP3-3, was amplified. The full-length cDNA was deduced from the genomic DNA sequence. Two primers, PbGPA3-F5 and PbGPA3-R5, including the start and stop codons, respectively, were designed and used to clone the full-length cDNA cPbGPA3-F5R5 from pDNR-cDNA library plasmids.

Cloning of the Gβ protein gene *GPB1* - Degenerated primers, Pbgpb-F1 and Pbgpb-R2 (Table S1), corresponding to conserved amino acid residues REAEXLKD and GDMTCMXW, respectively, were designed using homologous sequence information from Cpgb1 of *C. parasitica*

and SfaD of *A. nidulans* and other Gpb proteins of plant pathogenic fungi. A 300 bp fragment of the *PbGPB1* gene was obtained by PCR using genomic DNA as template. After sequencing, pair of specific primers, Pbgpb-F5 and Pbgpb-R5 (Table S1) were designed and a 150 bp fragment was amplified by PCR using our pDNR-cDNA library as the template. In order to clone the central part of the *GPB1* gene, Pbgpb-F5 and a degenerated primer Pbgpb-R11 were used to PCR amplify the Pbgpb-F5R11 fragment. After confirming the sequence of Pbgpb-F5R11, primers Pbgpb-F13, Pbgpb-F14, Pbgpb-R13 and Pbgpb-R14 were combined with primers M13F1, M13F2, M13R1 and M13R2 for amplification of 5'-termini and 3'-termini of the *GPB1* gene by nested PCR using our pDNR-cDNA library as template. After sequencing the fragments produced a pair of specific primers, PbGpb-NdeI-F and PbGpb-BanHI-R, were designed for amplification of the *GPB1* open reading frame from the pDNR-cDNA library plasmids.

Cloning of the Gγ protein gene *GPG1* - Degenerated primers Pbgpg-F2 and Pbgpg-R11 (Table S1) were designed based on the highly conserved regions, (MV)(AL)(DE)LK(LY)RR and D(TYNF)(ML)VP(SM)VWG, respectively, of Gγ protein homologies of human and plant pathogenic fungi in the GenBank database. A 152 bp fragment of the *PbGPG1* gene was amplified by PCR using our cDNA library as the template. After sequencing the fragment, primers Pbgpg-F5, Pbgpg-F6, Pbgpg-R3 and Pbgpg-R4 (Table S1) were combined with primers M13F1, M13F2, M13R1 and M13R2 for amplification of the 5'-termini and 3'-termini of the *GPG1* gene by nested PCR using cDNA as template. After sequencing the fragments produced; a pair of specific primers, PbGpg-NdeI-F and PbGpg-BanHI-R, were designed for amplification of the *GPG1* open reading frame from our pDNR-cDNA library.

Cloning of the *RAS2* gene - Degenerate primers Pbras-F1, Pbras-F2 and Pbras-R1 (Table S1) were designed based on the highly conserved GAGGVGKS and MTEYKLW regions, which correspond to the nucleotide binding site and N-terminus respectively, and the C-box domain found at the C-terminus of the G-protein family. These were used to amplify DNA fragments from

genomic DNA and subsequently from both our cDNA and genomic libraries. The sequences of the 5’ and 3’ UTRs were determined by genome walking.

Construction of a GPB1-flexible linker-GPG1 fusion protein - pGBKT7 and pGADT7 vectors were constructed for the expression of a GPB1-flexible linker-GPG1 fusion protein (Table 2). Essentially, the vector was constructed in the following manner. The *PbGPB1* ORF, without the stop codon, was PCR-amplified with primers, pbgpb-NdeI-F and Pbgpg-BamHI-R, using the cDNA of the *PbGPB1* gene as the template. The gene was ligated into *Nde*I and *BamH*I sites of pGADT-7, giving rise to pGADT-PbGPB1 (TAG). The polylinker (atgCGTATTAAAAACGGCAGCGGTGCGGCAGCCCCGAAAGCCGCTCCGGCCCTGACCC CGGCGGATGTGGCCCGCAGCtaa) was added before the 5’-termini of the *PbGPG1* gene. Firstly, part of the polylinker sequence was added into 5’-termini of the *PbGPG1* gene by PCR amplification with the primers pGADT-linker-gamma-F2 and pGADT-Gpb-R2, using the cDNA of the *PbGPG1* gene as the template. The resulting PCR product was then used as the template for the polylinker-*PbGPG1* construct in a second PCR reaction with the primers pGADT-linker-gamma-F2 and pGADT-Gpb-R2. The final PCR product was ligated into pGADT-PbGPG1 (TAG) at the *BamH*I site giving to pGADT-PbGPB1-polylinker-PbGPG1.

Identification of the ORF and transcript leaders - For each gene, the start codon ATG was determined by finding a Kozak consensus sequence (ANNATGG) at an ATG site and stop codons in the cDNA sequence upstream of it (Kozak, 1987 and Kozak, 1991); whilst a comparative analysis of the cDNA and genomic sequences was used to map introns within the open reading frame (ORF). Generally, transcript start sites were mapped by 5’-RACE RT-PCR and the poly(A) tail used to identify the end of the 3’ leader.

Nucleotide and protein sequence analysis – BLAST searches, using the ExPASy molecular biology server http://us.expasy.org/tools/blast/, were used to identify proteins that share sequence

similarity. Protein sequence alignments were performed with the Align program (VectorNTi, Informax). TATA-box elements were identified using the HC_tata program at http://125.itba.mi.cnr.it/~webgene/ wwwHC_tata.html. Transcriptional start sites were predicted using the Neurol Network Prediction Program at http://www.fruitfly.org/seq_tools/promoter.html. Gene sequences were screened for potential transcription factor binding elements using the program TFSEARCH at http://www.cbrc.jp/research/db /TFSEARCH.html.

Supplementary Data

Identification of the components of a cAMP signaling pathway in *P. brasiliensis* - In an attempt to identify genes from the cAMP-signaling pathway, which our studies clearly implicated in the control of the morphological switch from the mycelium to pathogenic yeast form of *P. brasiliensis*, we used homology based strategies to clone the genes that encode adenylate cyclase and several G-proteins, including ras, three Gα, Gβ and Gγ subunits. We found that in most cases the *P. brasiliensis* gene products had greatest similarity with the corresponding genes in *Aspergillus* species (Supplementary Fig. S1).

***Adenylate cyclase* –** the adenylate cyclase gene *CYR1* indicated an ORF of 6.3 kb that is interrupted by 3 introns at positions 170-341, 874-1332 and 6640-6713. It is predicted to encode a protein of 2100 amino acid residues with molecular weight of 234 kDa. An analysis of the adenylate cyclase protein sequence with SMART indicated that it contains 5 domains: a Gα association domain (GA, position 447-555); a Ras association domain (RA, position 562-643), 14 leucine-rich repeats (LRR_TYR domains, position 752-1244), a serine/threonine phosphatase family 2C catalytic domain (PP2Cc, position 1341-1627) and an adenylyl/guanylyl cyclase catalytic domain (CYCc domain, position 1574-1856).

Gα proteins **-** The Gα protein encoding gene *GPA1* consisted of a 1323 bp ORF, with 4 introns at positions 118-184, 526-584, 996-1065 and 1208-1283, which encoded a 353 amino-acid protein; the *GPA2* gene consisted of a 1394 bp ORF, with 4 introns at positions 130-208, 392-470, 633-709 and 967-1056, which encoded a 356 amino-acid protein; whilst the *GPA3* gene consisted of a 1489 bp ORF, with 4 introns at positions 127-269, 614-673, 801-864 and 1302-1390, which encoded a 359 amino-acid protein; and the three Gα proteins had 31.9% sequence identity. A phylogenetic analysis of all the fungal Gα proteins identified to date indicates that, with the exception of Gpa4 from *U. maydis*; they fall into three major families, each of which is represented by the three Gα proteins from *P. brasiliensis* (Fig. S1-D).

Gβ and Gγ proteins **-** In contrast to most fungal Gα proteins, FadA operates in conjunction with the Gβ protein SfaD (Rosen *et al.*, 1999). It occurred to us that if *P. brasiliensis* Gpa1 serves a related function as part of a conserved pathway, then it should posses a Gβ protein, which would have a high degree of sequence similarity to SfaD. Consistent with this proposal we identified a *P. brasiliensis* gene that encodes a Gβ protein. The *GPB1* gene consisted of a 1546 bp ORF, with 4 introns at positions 118-369, 1081-1172, 1261-1331 and 1450-1526, which encoded a 353 amino-acid protein, containing seven WD motifs, which has 94.3% identity to SfaD. This relationship appears to holds for other members of the Family I (Fig. S1-B), suggesting that these Gα proteins operate in conjunction with a Gβ protein. For several fungi, disruption of the Family I Gα protein and the corresponding Gβ protein affect the same pathways, consistent with an interaction of these proteins. In the case of *N. crassa* (Galagan *et al.*, 2003), *M. grisea* (Dean *et al.*, 2005) and *A. fumigatus* (Nieman *et al.*, 2005) the genome sequences have established that they possesses a single Gβ protein; and no more than one Gβ protein has been identified in any other fungi to date. Family II and III G_α proteins must either act alone, interact with the same Gβ protein or with as yet unidentified proteins. In *S. cerevisiae* Gpa2, which is a member of Family III, has been shown to interact with a kelch repeat protein rather than a Gβ-protein (Harashima and Heitman, 2002).

Recently, a Gγ protein, GpgA, was identified in *A. nidulans* (Seo *et al.*, 2005) and there is evidence that this forms a sfaD-GpgA dimer that can interact with FadA (Lafon *et al.*, 2005). Similarly we have identified a gene that encodes a Gγ protein, Gpg1, in *P. brasiliensis*. The *GPG1* gene consisted of a 498 bp ORF, with 2 introns at positions 156-304 and 363-437; which encodes a 91 amino-acid protein with 77.1% identity to GpgA from *A. nidulans* (Fig. S1-C). Furthermore, the *P. brasiliensis* Gpg1 possess a typical consensus sequence for C-terminal farnesylation that is present in other Gγ proteins. We have established that Gpa1, but not Gpa2 nor Gpa3, interacts with Gpb1 and Gpg1, which also interact with one another, indicative of the formation of a Gpa1-Gpb1-Gpg1 trimer.

***Ras* –** We only identified one *RAS* gene, although a number of degenerate oligonucleotides were used to screen for multiple genes, in *P. brasiliensis*. The *RAS* gene sequence indicates an ORF of 791 bp, which is interrupted by one intron at positions 78-164, that encodes a protein of 233 amino acid residues with a predicted molecular weight of 26.5 kDa. Over its entire length, the PbRas protein is 74.9% and 68.3% identical to RasB and Ras2, but only 46.4% and 42.3% identical to RasA and Ras1, from *A. fumigatus* and *N. crassa*, respectively (data not shown). A recent *P. brasiliensis* EST-sequencing project has also identified the same *RAS* gene, termed *RAS2*, but not a second gene (Felipe *et al.*, 2005).

Comparison of the gene transcripts - The *CYR1* gene had a 907 bp 5’-leader that incorporated several poly(C) motifs, which could be binding-sites for poly(C)-binding proteins (PCBPs) that bind mRNAs to stabilize them or silence their translation (Makeyeve *et al.*, 2002). There is a putative polyadenylation-signal (e.g. ATAAA) 7 bp downstream of the stop codon but the 3’-leader, as defined by the start of the polyadenylate sequence of the cDNA, is 603 bp long. The presence of such an extensive 3’-leader suggests that it may serve as a post-transcriptional regulatory element. Indeed, we note that it is extremely U-rich, a property that may serve to target it to poly(U)-binding proteins (PUBPs), which stabilize transcripts (Duttagupta *et al.*, 2005).

However, it also contains six copies of the AUUUA consensus motif found in mammalian AU-rich response elements (ARE), which target ARE-binding proteins that destabilize transcripts (Ross, 1995). In contrast, there are none of these sequences in the much larger 5'-leader. Interestingly, the first intron is differentially spliced to produce three transcripts that encode adenylate cyclase proteins that are shorter by 1 and 7 amino acid residues, but we do not know if these are functionally different.

The transcripts for the three *GPA* genes differed in that *GPA1* and *GPA3* had relatively short 5'-leaders, consisting of 128 and 31 bp respectively, compared to *GPA2* that had a 2294 bp 5'-leader. Moreover, five introns were spliced-out of the 5'-UTR, at positions 278-230, 431-315, 1585-1518, 1894-1802, and 2131-2066 bp upstream of the conceptual ATG, to produce the 5'-leader for the *GPA2* transcript. Interestingly, this leader possessed nine AUUUA motifs, four of which occur consecutively between positions -2498 and -2517, and ten poly(A) (e.g. A_5 or A_6) motifs. The *GPA1* transcript, in contrast to *GPA2* and *GPA3*, has a substantial 3'-leader of 554 bp that is U-rich, with eleven poly(U) motifs (e.g. U_5-U_9), which might target it to PUBPs. The *GPB1* transcript is 3.3 kb, with a 5'-leader of 577 bp; whilst a putative polyadenylation signal (e.g. AAATTA), 12 bp downstream of the stop codon, marks the start of a long 3'leader of 1171 bp, which terminates with a poly(A) tail. The only notable feature of the 5'-leader was the presence of 3 poly(C) sequences (e.g. C_5), but these were not as extensive or prevalent as those found in the 5'leader of *CYR1*; whilst the 3'-leader is U and A-rich, with these bases arranged into poly(U) (e.g. U_5 or U_6) and poly(A) (e.g. A_5) sequences, but with only one AUUUA sequence, which may serve to target the transcript to poly(U) (PUBP) and poly(A) (PABP) binding-proteins. The *GPG1* transcript is 1.134 kb and incorporates a 370-bp 5'-leader, which is produced when an 87-bp intron is spliced out of the 5'UTR at a position 99 bp downstream of the start-codon, and a 484-bp 3'-leader that possessed two poly(U) sequences (e.g. U_6). It was recently reported that a 315-bp intron is spliced-out of the 5'UTR of the *gng-1* gene that encodes a Gγ protein in *N. crassa* and that this is also a feature of mammalian genes that encode Gγ subunits (Krystofova and Borkovich, 2005). The splicing of

introns from the 5' UTR of *GPA2* and *GPG1* suggests that the expression of these genes is subject to a further level of regulation. Previously, we have shown that the expression of the *P. brasiliensis HSP70* gene was subject to regulation by intron splicing, in that there was an accumulation of unspliced transcripts in mycelium but not yeast (Petrofeza da Silva *et al.*, 1999).

References

Binz T, Jalali F, Horgen PA. (1998) Isolation of adenylate cyclase gene-specific sequences from *Ophiostoma novo-ulmi*, *Candida albicans*, and *Agaricus bisporus* by PCR. Curr Microbiol. 37, 359-361

Dean RA, Talbot NJ, Ebbole DJ, Farman ML, Mitchell TK, Orbach MJ, Thon M, Kulkarni R, Xu JR, Pan H, Read ND, Lee YH, Carbone I, Brown D, Oh YY, Donofrio N, Jeong JS, Soanes DM, Djonovic S, Kolomiets E, Rehmeyer C, Li W, Harding M, Kim S, Lebrun MH, Bohnert H, Coughlan S, Butler J, Calvo S, Ma LJ, Nicol R, Purcell S, Nusbaum C, Galagan JE, Birren BW. (2005) The genome sequence of the rice blast fungus *Magnaporthe grisea*. Nature 434, 980-986.

Duttagupta R, Tian B, Wilusz CJ, Khounh DT, Soteropoulos P, Ouyang M, Dougherty JP, Peltz SW. (2005) Global analysis of Pub1p targets reveals a coordinate control of gene expression through modulation of binding and stability. Mol Cell Biol. 25, 5499-5513.

Felipe MS, Andrade RV, Arraes FB, Nicola AM, Maranhao AQ, Torres FA, Silva-Pereira I, Pocas-Fonseca MJ, Campos EG, Moraes LM, Andrade PA, Tavares AH, Silva SS, Kyaw CM, Souza DP, Network P, Pereira M, Jesuino RS, Andrade EV, Parente JA, Oliveira GS, Barbosa MS, Martins NF, Fachin AL, Cardoso RS, Passos GA, Almeida NF, Walter ME, Soares CM, Carvalho MJ, Brigido MM. (2005) Transcriptional Profiles of the Human Pathogenic Fungus *Paracoccidioides brasiliensis* in Mycelium and Yeast Cells. J Biol Chem. 280, 24706-24714.

Galagan JE, Calvo SE, Borkovich KA, Selker EU, Read ND, Jaffe D, FitzHugh W, Ma LJ, Smirnov S, Purcell S, Rehman B, Elkins T, Engels R, Wang S, Nielsen CB, Butler J, Endrizzi M, Qui D, Ianakiev P, Bell-Pedersen D, Nelson MA, Werner-Washburne M, Selitrennikoff CP, Kinsey JA, Braun EL, Zelter A, Schulte U, Kothe GO, Jedd G, Mewes W, Staben C, Marcotte E, Greenberg D, Roy A, Foley K, Naylor J, Stange-Thomann N, Barrett R, Gnerre S, Kamal M, Kamvysselis M, Mauceli E, Bielke C, Rudd S, Frishman D, Krystofova S, Rasmussen C, Metzenberg RL, Perkins DD, Kroken S, Cogoni C, Macino G, Catcheside D, Li W, Pratt RJ, Osmani SA, DeSouza CP, Glass L, Orbach MJ, Berglund JA, Voelker R, Yarden O, Plamann M, Seiler S, Dunlap J, Radford A, Aramayo R, Natvig DO, Alex LA, Mannhaupt G, Ebbole DJ, Freitag M, Paulsen I, Sachs MS, Lander ES, Nusbaum C and Birren B. (2003) The genome sequence of the filamentous fungus *Neurospora crassa*. Nature 422, 859-868.

Harashima T, Heitman J. (2002) The Gα protein Gpa2 controls yeast differentiation by interacting with kelch repeat proteins that mimic Gβ subunits. Mol Cell. 10, 163-173.

Kozak M. (1987) At least six nucleotides preceding the AUG initiator codon enhance translation in mammalian cells. J Mol Biol. 196, 947-950.

Kozak M. (1987) An analysis of vertebrate mRNA sequences: intimations of translational control. J Cell Biol. 115, 887-903.

Krystofova S, Borkovich KA. (2005) The heterotrimeric G-protein subunits GNG-1 and GNB-1 form a Gbetagamma dimer required for normal female fertility, asexual development, and galpha protein levels in *Neurospora crassa*. Eukaryot Cell 4, 365-378.

Lafon A, Seo JA, Han KH, Yu JH, d'Enfert C. (2005) The heterotrimeric G-protein GanB({alpha})-SfaD({beta})-GpgA({gamma}) is a carbon source sensor involved in early cAMP-dependent germination in *Aspergillus nidulans*. Genetics 171, 71-80.

Makeyev AV, Liebhaber SA. (2002) The poly(C)-binding proteins: a multiplicity of functions and a search for mechanisms RNA 8, 265–278.

Nierman WC, Pain A, Anderson MJ, Wortman JR, Kim HS, Arroyo J, Berriman M, Abe K, Archer DB, Bermejo C, Bennett J, Bowyer P, Chen D, Collins M, Coulsen R, Davies R, Dyer PS, Farman M, Fedorova N, Fedorova N, Feldblyum TV, Fischer R, Fosker N, Fraser A, Garcia JL, Garcia MJ, Goble A, Goldman GH, Gomi K, Griffith-Jones S, Gwilliam R, Haas B, Haas H, Harris D, Horiuchi H, Huang J, Humphray S, Jimenez J, Keller N, Khouri H, Kitamoto K, Kobayashi T, Konzack S, Kulkarni R, Kumagai T, Lafon A, Latge JP, Li W, Lord A, Lu C, Majoros WH, May GS, Miller BL, Mohamoud Y, Molina M, Monod M, Mouyna I, Mulligan S, Murphy L, O'Neil S, Paulsen I, Penalva MA, Pertea M, Price C, Pritchard BL, Quail MA, Rabbinowitsch E, Rawlins N, Rajandream MA, Reichard U, Renauld H, Robson GD, Rodriguez de Cordoba S, Rodriguez-Pena JM, Ronning CM, Rutter S, Salzberg SL, Sanchez M, Sanchez-Ferrero JC, Saunders D, Seeger K, Squares R, Squares S, Takeuchi M, Tekaia F, Turner G, Vazquez de Aldana CR, Weidman J, White O, Woodward J, Yu JH, Fraser C, Galagan JE, Asai K, Machida M, Hall N, Barrell B, Denning DW. (2005) Genomic sequence of the pathogenic and allergenic filamentous fungus *Aspergillus fumigatus*. Nature 438, 1151-1156.

Petrofeza da Silva S, Borges-Walmsley MI, Pereira IS, Soares CM, Walmsley AR, Felipe MS. (1999) Differential expression of an *hsp70* gene during transition from the mycelial to the infective yeast form of the human pathogenic fungus *Paracoccidioides brasiliensis*. Mol. Microbiol. 31, 1039-1050.

Ross, J. (1995) mRNA stability in mammalian cells. Microbiol Rev. 59, 423-450.

Rosen S, Yu JH, Adams TH. (1999) The *Aspergillus nidulans sfaD* gene encodes a G protein beta subunit that is required for normal growth and repression of sporulation. EMBO J. 18, 5592-5600.

Supplementary Figures

Fig. S1. The phylogenetic relationship between *Paracoccidioides brasiliensis* and other fungal cAMP-signaling proteins - A neighbour-joining bootstrap tree is derived from the amino acid sequences alignments of fungal (A) adenylate cyclase (AC) (B) Gβ, (C) Gγ and (D) Gα proteins, using the VectorNTi 6.0 align program (Informax). The GenBank accession numbers of these proteins are indicated following the abbreviated names of the proteins. The abbreviations are as follows: Pb, *P. brasiliensis*; Af, *Aspergillus fumigatus;* An, *A. nidulans*; Bf, *Botryotinia fuckeliana;* Bg, *Blumeria graminis*; Ca, *Candida albicans;* Cc, *Coprinellus congregatus;* Ch, *Cochliobolus heterostrophus;* Cn, *Cryptococcus neophormans*; Cp, *Cryphonectria parasitica*; Ct. *Colletotrichum trifolii;* Fo, *Fusarium oxysporum*; Gz, *Gilbberella zeae;* Hs, *Homo sapiens*; Kl, *Kluyveromyces lactis;* Le, *Lentinula edodes*; Ma, *Metarhizium anisopliae*; Mg, *Magnaporthe grisea*; Nc, *Neurospora crassa*; Oy, *Oculimacula yallundae;* Pa, *Podospora anserine;* Pm, *Penicillium marneffei;* Rn, *Rosellinia necatrix;* Sc, *S. cerevisiea*; Sk, *Saccharomyces kluyveri*; Sp, *Schizosaccharomyces pombe*; Ss, *Sporothrix schenckii;* Ta, *Trichoderma atroviride*; Uh, *Ustilago hordei;* and Um, *Ustilago maydis*; Zm, *Zea mays*. The Gα proteins from *P. brasiliensis* are indicated on the diagram as PgGpa1, PbGpa2 and PbGpa3. The GenBank accession numbers for the *P. brasiliensis* proteins are as follows: Cyr1 (AAS01025), Gpa1 (AAT40562), Gpa2 (AAT40564), Gpa3 (AAT40563), Gpb1 (AAT40565) and Gpg1 (EF687895).

Fig. S2. The *RAS* transcript levels during the mycelium to yeast transition. A bar chart showing the *RAS* transcript levels at the indicated times following an increase in temperature from 26°C to 37°C to induce the mycelium-to-yeast transformation. The data represent the average of 3 independent measurements. The measured quantity of the *P. brasiliensis RAS* gene mRNA in each of the treated samples was normalized by using the C_T values obtained for the *α-tubulin* RNA amplifications run on the same plate. The relative quantitation of the *RAS* and *α-tubulin* gene expression was determined by a standard curve (i.e., C_t values plotted against the

logarithm of the DNA copy number). The values represent the number of copies of the cDNAs of the *RAS* gene divided by the number of copies of the cDNAs of the α-*tubulin* gene.

Supplementary Tables

Table S1: Primers used for gene cloning.

**Primers**	**Sequences**<br>(5'→3'; restriction sites are underlined; A+C+G=V, T+C+G=B, A+T+G=D, A+T+C=H, A+T=W, C+G=S, T+G=K, A+C=M, C+T=Y, A+G=R, A+T+C+G=N)
PbCYR1	
PbAC-F1	GTBTTCACYGAYATYAAGAACWSBAC
PbAC-F2	AGAACTGCACGTCGCTTTGG
PbAC-F3	GGAAGCTGAAGGGGCTTGAG
PbAC-F4	AGTTGCGGATGGTGGGCAAATC
PbAC-F5	CCGTCCTCGAGGTGCAATGGAAACCCCATCCTCG
PbAC-F6	TATGGCAGATAGCCTTGGGCGAAAC
PbAC-F7	CAATCTGAAGAGTCTCGTTGATCTTGATATC
PbAC-F8	GTGCCCTGAAAATTTCGGAGAGATTGGTCTTC
PbAC-F9	GTCTGGCGCCGTTGGACTGTTGCTTTACGTTTGG
PbAC-F10	GCCTCAACGCTCCACGGGCTGAGATTGAAGAGG
PbAC-F11	CCGTTGCCTATGACTTGATGTC
PbAC-F12	CCAGACACTCGTCGTCCTCAAAGAGTA
PbAC-F14	GAGAATTCCGCAATCCCTTTGAACCCTCCAG
PbAC-F15	CGTTTACGTTTGCGGGATCTCTGAAGCAAC
PbAC-F16	GAATATCGGCCGGATGAATCACATCCACTC
PbAC-F17	AGCGGCCAGAAACTTAGATTTGCGGCAGCA
PbAC-F18	CAGCATGGCAAGGAGACAGCGGGAGAAAGA
PbAC-F19	GAGATGCACCTGTGTATCCAGTTCCAGGAGA
PbAC-F20	CCCCAACCCGGGATTTGAGCTACTCACAGA or<br>TCCACGTCGCTTTGGGAGACGTATCC
PbAC-F21	TTGGGATACCGTCTGCTCTTCCTCTCGCCA
PbAC-F22	AGAGAGTCCCGTGCGCTGACACAGCCTTGA
PbAC-F23	TTGTCACCGCGAGTTAAAGTCCGGAGTTGA
PbAC-F24	CCACGTCGACTTGCACGGGCGCAGTCTCA
PbAC-F25	GTCATATCTCGAGCTGCAACTGAAACCCCA
PbAC-F26(KpnI)	<u>GGTACC</u>AAAATGTCTAGGAGACAGCGGGAGAAAGATAGG
PbAC-F27	AGATTTCGGATCAGGATGACAGAAGGCTGA
PbAC-F28	GATTTCGGATCAGGATGACAGACACTCGTC
PbAC-F40	CCGCATTTCCGCAGTTGCGGATGGTGGGCAAATC
PbAC-GSP1	TGATGCTGTTGAATCGTGCGTCTATCTC
PbAC-R1	TTVACCATRGGRCCSWRGATRTCCAT
PbAC-R2	CAAGTAATCCATTCGACCAG
PbAC-R3	CCGTCAACTCGTTATAGGACAAG
PbAC-R4	NNNNCTCGAGTGGCGGCCGCTCTAGAACTA
PbAC-R5	NNNNCTCGAGGTCTATAATATCTAATAGTGATG
PbAC-R6	CAGGATTCTCAAGCCCCTTCAGCTTC
PbAC-R7	AGTCACCCATAACCGTTCCAGTGTCGTAAGGCGTC
PbAC-R8	TTCCAGTGTCGTAAGGCGTCCAATATCCGACAACTC
PbAC-R9	ACTCACCTCCACCCGCGTAACCTGATGTTC
PbAC-R9(2)	GTTGAAGCTGATATCAAGATCAACGAGACTCTTC
PbAC-R10	GTCCAGAGTTCTTGAAAGGTC
PbAC-R11	CGCAGACTCCTTTTTGCGGTCAGACTCGACAC
PbAC-R12	AGCTCGAATTCCGACCATATCTAGCAGGAGAC
PbAC-R13	TCAAAAACCTAGATCCCTTCCCACCACCGTCG
PbAC-R14	GATTGACTGCCAGATGACATAGG

PbAC-R15	GTGGTGTCCATGATGGCGACCGCTTCC
PbAC-R16	GTCATCCGATGCGCCAAGAATCGCAAG
PbAC-R17	CCAAAGCTGGTTGTCGGTACGTCTGTTG
PbAC-R18	CAGAGGAGAGAACTTGGCATTTCCGAACCA
PbAC-R19	TATCTGAGCCAACTGTCGACGGAAGAGATC
PbAC-R20	TCCAAAGCTGGTTGTCGGTACGTCTGTTGG
PbAC-R21	TGGGGATACATCGGGGGAATGTGGCCTAC
PbAC-R22	GAGTCACGCTCGCCATCTGTCATGGAACCA
PbAC-R23	CTCTTTGAGGACGACGAGTGTATTTTGAAC
PbAC-R24	AAGAAACCCCCAAGCAGCATGAAGCAGGCA
PbAC-R25	ACCCATCCAAGCCCGAAAGAGCAACGTCCA
PbAC-R26	GCCAAACAGCGAAACAACGCCGTTCCGGCA
PbAC-R27	GGCGTTACGATTACTCTTTGAGGACGACGA
PbAC-R28	GAATGTGGCCTACGGGGGCGTTACGATTA
PbAC-R29	CTGGTTGTCGGTACGTCTGTTGGGGATACA
PbAC-R30	CCCTGCGGAATCTCCGTCAACTCGTTATAGGACAAG
PbAC-R31	GACGGGGGGTTGAAGCTTTCGCCTCTCACA
PbAC-R32	AACAATATCGATCGTACCAAGCAAACAGACGCA
PbAC-R33	CAAATCCACCCATATGAGCCAACTGTCGAC
PbAC-R330	CATACTGTGGCCTGCAAGCAGGAGATTCCA
PbAC-R34(NotI)	GCGGCCGCGCCGTGCTCGAAGAACTAGAACCAC
PbAC-R35	CTCCATAGTTTGATATATCCTTTGATTGAC
PbAC-R36	CCATAGTTTGATATATCCTTCAGAAGACTTTCCG
PbAC-R37	CATTCATAAAGCAGCTGTTCCTGTGGCAGG
PbGPA1	
PbGPA-DGF5	GCYGGWGARYSNGGNAARTCNAC
PbGPA-DGR2	CARTGDATCCAYTTYTTNCK
PbGPA-DGF5R2-3-4-F1	GAACAGCATGCCACTCATAGC
PbGPA-DGF5R2-3-4-R1	TCTAACGGCTCTGTCACTTGG
PbGPA-DGF5R2-3-2-F1	GAGCTTTGCAAGGCTTAACTCG
PbGPA-DGF5R2-3-2-R1	ATCGTGGCTCACGCATTTCGAG
PbGPA1-ExGPAF1	CATATGGGTTGTGGAATGAGC
PbGPA-F1	CGGGATCCGTCATATGGGTTGTGGAATGAGCACC
PbGPA1-F1	TGTCCTTCGCTCCCGTGTTAAGACCACAGG
PbGPA1-F2	TTGCGACTATATCCTCAACCGCTTCGTCTCGCTC
PbGPA1-F3	GACTATATCCTCAACCGCTTCGTCTCGCTCAACC
PbGPA1-F4	GATTGATCGGTTTAAGGAGAAACTGCCTGTTAGC
PbGPA1-F5	CTCTGAGTACGACCAGCTCCTGTTCGAAGACG
PbGPA1-F6	GACATGAAGCAGAACCTGTACATTCCGATATCGTC
PbGPA1-F7	GTGTCATTCATCCATCTTGCTTTACGCC
PbGPA1-F8	GGCTATGGAAGGACTAGATATCCCGTTGGA
PbGPA1-F9	CTGCATGGTTTCCTTCTCAG
PbGPA1-F10	GATCAAAATGCTCTTGCTTGGTGCTGGAGA
PbGPA1-F11	GAATGACTCTGCACGATACTATTTTGACTCCA
PbGPA1-R1	CGGGATCCTCATATCAGTCCACAGAGGCGAAG
PbGPA1-R2	TCCAACGGGATATCTAGTCCTTCCATAGCC or GCTTAGCCTAAACTCTCCATAGTCATCGTGTCC
PbGPA1-R3	GAGCATTTTGATCTCGTTTCTCTGCAGC
PbGPA1-R4	TCCTTGTCCTCGGTGCTCATTCCACACC
PbGPA1-R5	CCTGTGGTCTTAACACGGGAGCGAAGGACA
PbGPA1-R6	TGAAGACTGGCTGGGTTTTGCGGGAAAGGA
PbGPA1-R7	CAATTACCCATGGCTTATTAGG
PbGPA1-R8	CAGTGTATTCGGCACCCCCTTCGTAATCAG
PbGPA1-R9	TGTCCTGGATGATTATATCGTTGACAGCCA
PbGPA1-pYER3	GCCTAGGTCATATCAGTCCACAGAGGCGAAG

PbGPA2	
PbGPA2-F1	ACGAACGATACCAAAACCGCGCGGTCATCTACTC
PbGPA2-F2	ACCGCGTCTTTACCCCTGGGTGGTTACCCA
PbGPA2-F3	GGAGATCTACATCCACTACACAAATGCTAC
PbGPA2-F4	TCAATGAGTGCAAACGGGATCTGCAAGGGA or<br>CATGGGTTGCGCAAGTTCGAACGGTGAAGA
PbGPA2-F5	CGCACTGGATGCCACTGGAAGGTGGCTTGA
PbGPA2-F6	CATGGGTTGCGCAAGTTCTCAACCAGTGGA
PbGPA2-F7(EcoRI)	CTAGGAATTCATGGGTTGCGCAAGTTCTCA
PbGPA2-F8	GGTGAAGATTCTTTTGCTTGGTGCTGGAGA
PbGPA2-F9	TTGAGTTTGAAAAGACAAAGCCACTAGCCA
PbGPA2-F10(BD-NdeI)	ATGGCCATGGAGGCCGAACATATGGGTTGC
PbGPA2-R1	TGAAGCATGTCTTGATTGTTGGGTAACCACC
PbGPA2-R2	ATCGAGGAGAATCTTGAATGCAACAACCATG
PbGPA2-R3	GCCGTCGACCACGCGTGCCCTATAGTGA
PbGPA2-R4	CATACGGATCATCTTGTCGATCGCAGACCA
PbGPA2-R5	TGGTGCAGAAACCGAGCGTGGCACAAGAGC
PbGPA2-R6	CTGTTGGCACCTACAGAATCAGGTTGTTGA
PbGPA2-R7	CAATTCCTATCAGAACTCAATCAGCATGCA
PbGPA2-R8	AACGTAGCTCGAGTCCTTCCCCTCTGTTCA
PbGPA2-R9	CTTCGCACTTTTCAAATGCCACGGGACATG
PbGPA2-R10	GGCTTCATGCATTTGATTTGCATTTTGATCC or<br>GAGCACAACTCTCAAGTGAGTGCAACACCA
PbGPA3	
PbGPA3-F1	TCTTGGATTGCGCCAAGGACCTGATAGGAG
PbGPA3-F2	CCTAATGAAGCCGATGTGTTGCGCGCTAGA
PbGPA3-F3	TATCCTCAGTATGTATAGGAAAAGCACACC
PbGPA3-F4	AACCGTGCCGCCAAGTACTTGCTCTGGAGA
PbGPA3-F5	GTATCAGATACCTACCCACCCACAACGT or<br>TGGTATCAGATAGGATGGGTGGGTGTTGCA
PbGPA3-F6	GTGGGTGTTGCAGTTCTGCTTCTGGGGAGA
PbGPA3-F7	CCACTTGGCCTTTTTTCCTTTTCCTTTTCC
PbGPA3-F8	CAGCTCTGGTCCACTTGGCC
PbGPA3-F8(EcoRI)	ATCAGAATTCATGGGTGGGTGTTGCAGTTC
PbGPA3-F9	ATGCAAGATCCTACTGCTTGGTTCGGGTGA
PbGPA3-F10	TGGATTCAGCACCTTACTTCTTCGAGGAAGC
PbGPA3-F11(BD-NdeI)	ATGGCCATGGAGGCCGAACATATGGGTGGG
PbGPA3-R1	TTCACTCCGCTGCCCGCCAACATCGAAC
PbGPA3-R2	ACGCACTGAATACTCAATTGGCCCATTGTG
PbGPA3-R3	GCGTATTCGGGTCCGCGTCAATCTGGTATG
PbGPA3-R4	CCTCTGCTCTGCATTCTCCCCAGAAGCAGA
PbGPA3-R5	CCCTTTCACAAAATACCAGAATCTTTCAGG
PbGPA3-R6	AAAGCAGTGAGTGGCCTGGGGAGTTATACG
PbGPA3-R7	GATTTGATAAGTAAGCGGCCCGATGCGGGA
PbGPA3-R8	GTCGGTGGCTTGAGTAAGGTGAGGATAAAG
PbGPB1	
PbGPB-F1	GCNAARATHTAYGCNATGCAYTGG
PbGPB-F2	TGGGTNATGACNTGYGCNTAYGC
PbGPB-R1	GCRTANGCRCANGTCATNACCC
PbGPB-R2	CCABWKCATRCANGTCATRTCNCC
Pbgpb-F5	TATGCGATGCACTGGTCGACAG
Pbgpb-R5	CAGGTCATGACCCATGACGAT
Pbgpb-R11	GCCCAGAYYTTNAGCARNGARTCCCA

Pbgpb-F13	CTTCCGTTGCGTTCTCAGTCTCTGGTCG
Pbgpb-F14	CGTGTCAGCTGCCTGGGCGTCAGCAAC
Pbgpb-R13	GTCTAGACCACCACAGGCGACATAGTTACC
Pbgpb-R14	GAAACCAGATGGCGGCGGTCTGTCG ACC
pbgpb-NdeI-F	ACGCTCATATGGCGGCCGATTTGAGCGG
pbgpb-BanHI-R	CGATGGATCCCTACCATGCCCAGACCTTGAG
PbGPG1	
Pbgpg-F2	RTGKCNGABCTVAARCTSCGNMGR
Pbgpg-R11	NCCCCANACVSWNGGNACCAKRWRGTC
Pbgpg-F5	GATCTAGAAAGACCGCGCGTAAAGGTGTCTG
Pbgpg-F6	GACCGCGCGTAAAGGTGTCTGAGGCGGCCATG
Pbgpg-R3	CATGGCCGCCTCAGACACCTTTACGCGCGGTC
Pbgpg-R4	CAGACACCTTTACGCGCGGTCTTTCTAGATC
Pbgpg-NdeI-F	CATATGGCCCCTGCCTACGAGCTTCGAC
Pbgpg-BamHI-R	GGATCCTTACATGATCATACAGCAGCCACCTGAT
PbRAS2	
Pbras-F1	GGGGCTGGSGGSGTSGGGRAA
Pbras-F2	GATGACAGAATATAAACTCGTSGTSGT
Pbras-R1	CTCAAAGAAGAATTAGACACTTC
**Universal primers**	
AP1	GTAATACGACTCACTATAGGGC
AP2	ACTATAGGGCACGCGTGGT
AP11	GTAATACGACTCACTATAGGGCACGC
AP21	TCACTATAGGGCACGCGTGGTCGAC
AP3	TACGACTCACTATAGGGCACGCGTGGTCGA
AP4	GTAATACGACTCACTATAGGGCACGCGTGG
CDS/3’BamHI(2)	ATTCTAGACGCGGATCCGCGGACATGTTTTTTTTTTTT<br>TTTTTTTTTTTTTTTTTTVX
CDS/3’BamHI(3)	GATTCTAGACCGGATCCGCGGACATGTTTTTTTTTTTT<br>TTTTTTTTTTTTTTTTTTVN
CDSIII/3’BamHI	AACGCGGATCCGCGTTTTTTTTTTTTTTTTTTTTTTTT<br>TTTTT
Creator-F1	CGACGGTACCGGACATATGCCCGGGAATTC
Creator-R1	GCGCGCCAAACGAATGGTCTAGAAAGCTTC
M13F1	ACCGCCTTACGCGTGTAAAACGACGGCCAG
M13 forward	GCGTGTAAAACGACGGCCAG
M13R1	CCAGGATCTCCTAGGGAAACAGCTATGACC
M13 reverse	GGGAAACAGCTATGACCATG
PolyT	TTTTTTTTTTTTTTTTTTTTTTTTTVN
5’RACE	AAGCAGTGGTATCAACGCAGAGT
3’RACE	ATTCTAGAGGCCGAGGCGGCCGACAT
SIV1	AAGAAGTGGTATCAACGCAGAGTGGCCATTACG
SIV2	GTATCAACGCAGAGTGGCCATTACGGCCGG
SIVBamHI	AAGCAGTGGTATCAACGCAGAGTGGATCCGGG
Smart IV	AAGCAGTGGTATCAACGCAGAGTGGCCATTACGGCC<br>GGG

Supplementary Fig. S1

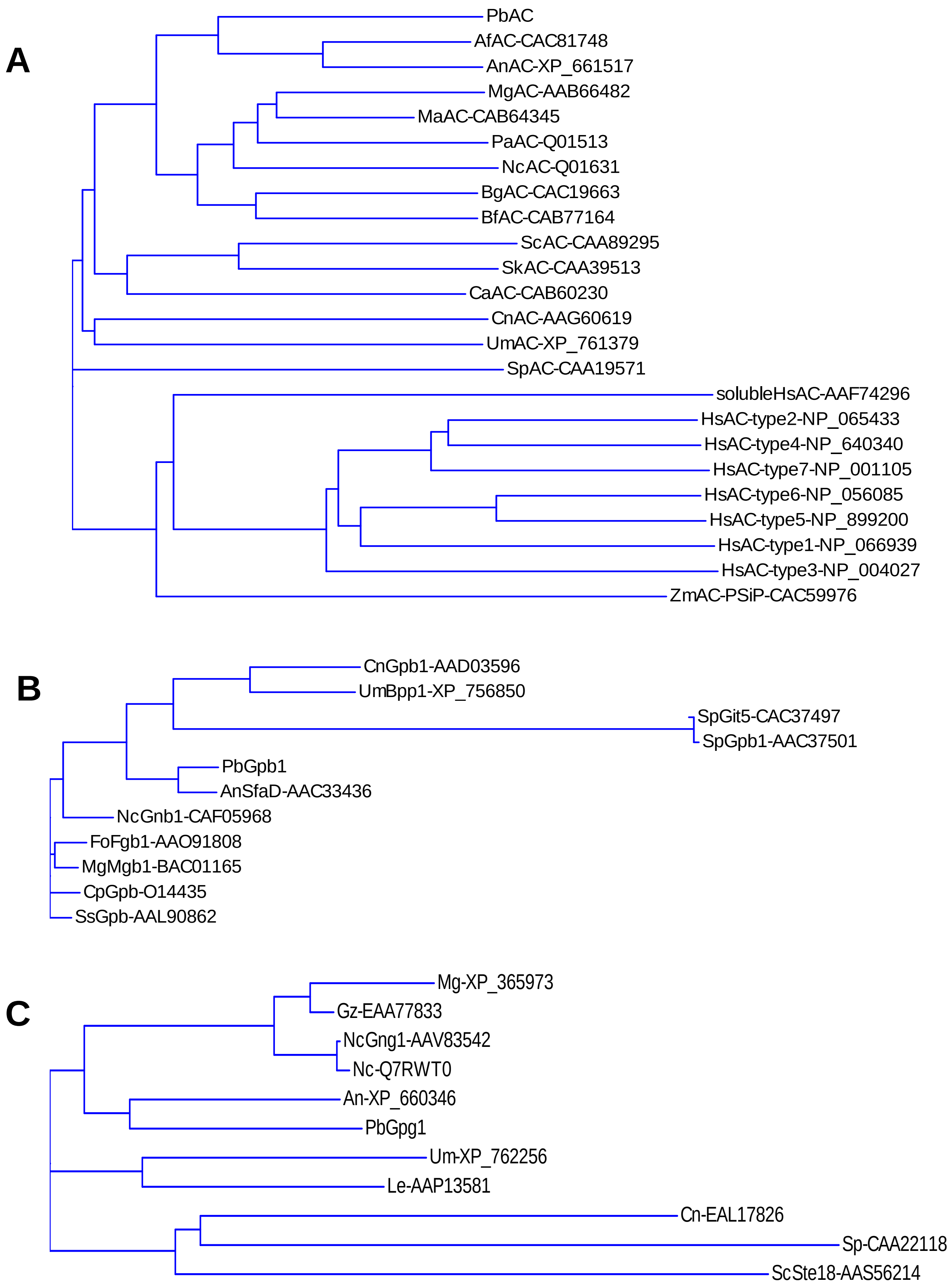

D

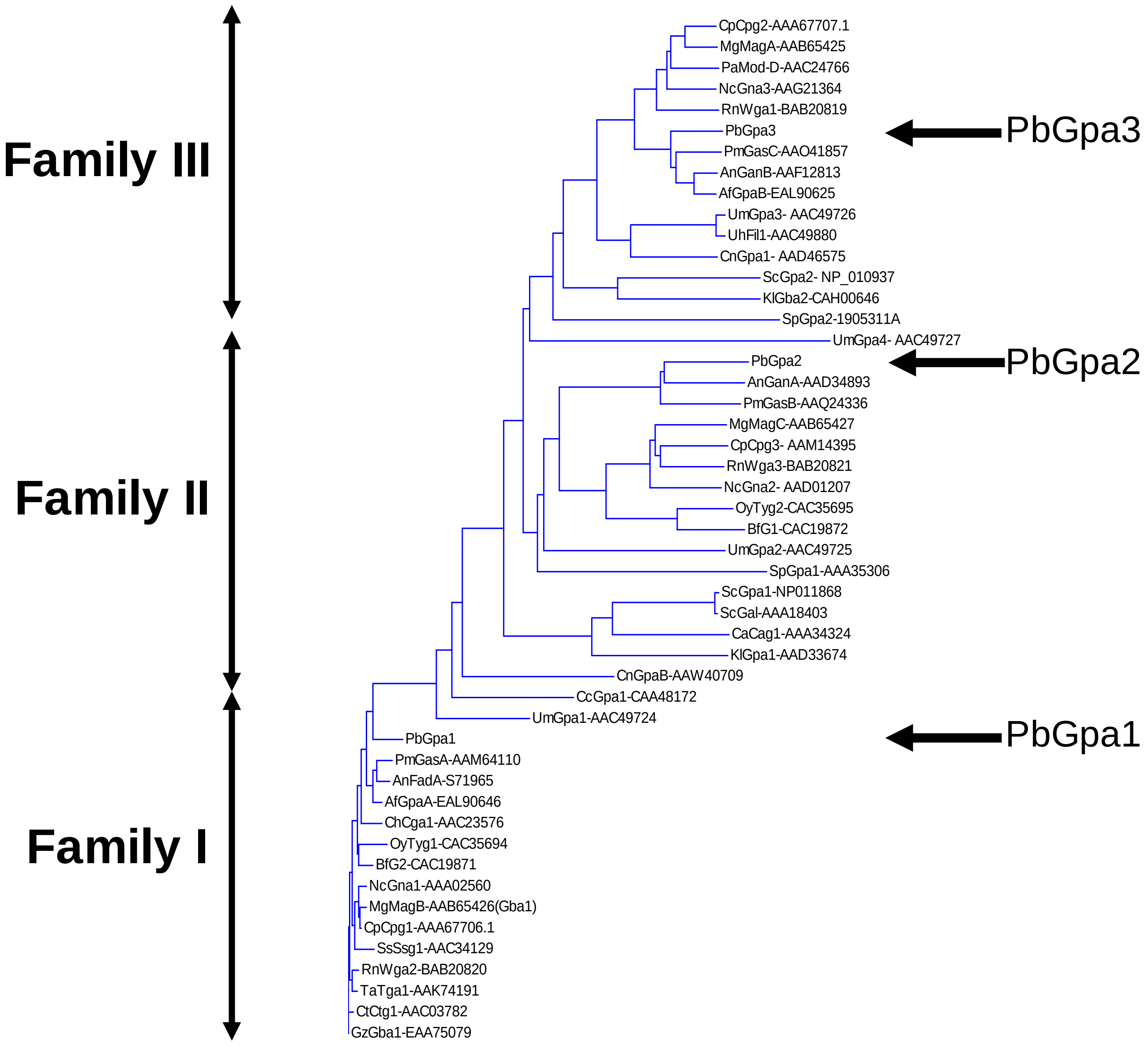

Family III
Family II
Family I
CpCpg2-AAA67707.1
MgMagA-AAB65425
PaMod-D-AAC24766
NcGna3-AAG21364
RnWga1-BAB20819
PbGpa3
PmGasC-AAO41857
AnGanB-AAF12813
AfGpaB-EAL90625
UmGpa3- AAC49726
UhFil1-AAC49880
CnGpa1- AAD46575
ScGpa2- NP_010937
KlGba2-CAH00646
SpGpa2-1905311A
UmGpa4- AAC49727
PbGpa2
AnGanA-AAD34893
PmGasB-AAQ24336
MgMagC-AAB65427
CpCpg3- AAM14395
RnWga3-BAB20821
NcGna2- AAD01207
OyTyg2-CAC35695
BfG1-CAC19872
UmGpa2-AAC49725
SpGpa1-AAA35306
ScGpa1-NP011868
ScGal-AAA18403
CaCag1-AAA34324
KlGpa1-AAD33674
CnGpaB-AAW40709
CcGpa1-CAA48172
UmGpa1-AAC49724
PbGpa1
PmGasA-AAM64110
AnFadA-S71965
AfGpaA-EAL90646
ChCga1-AAC23576
OyTyg1-CAC35694
BfG2-CAC19871
NcGna1-AAA02560
MgMagB-AAB65426(Gba1)
CpCpg1-AAA67706.1
SsSsg1-AAC34129
RnWga2-BAB20820
TaTga1-AAK74191
CtCtg1-AAC03782
GzGba1-EAA75079
PbGpa3
PbGpa2
PbGpa1

Supplementary Fig. S2

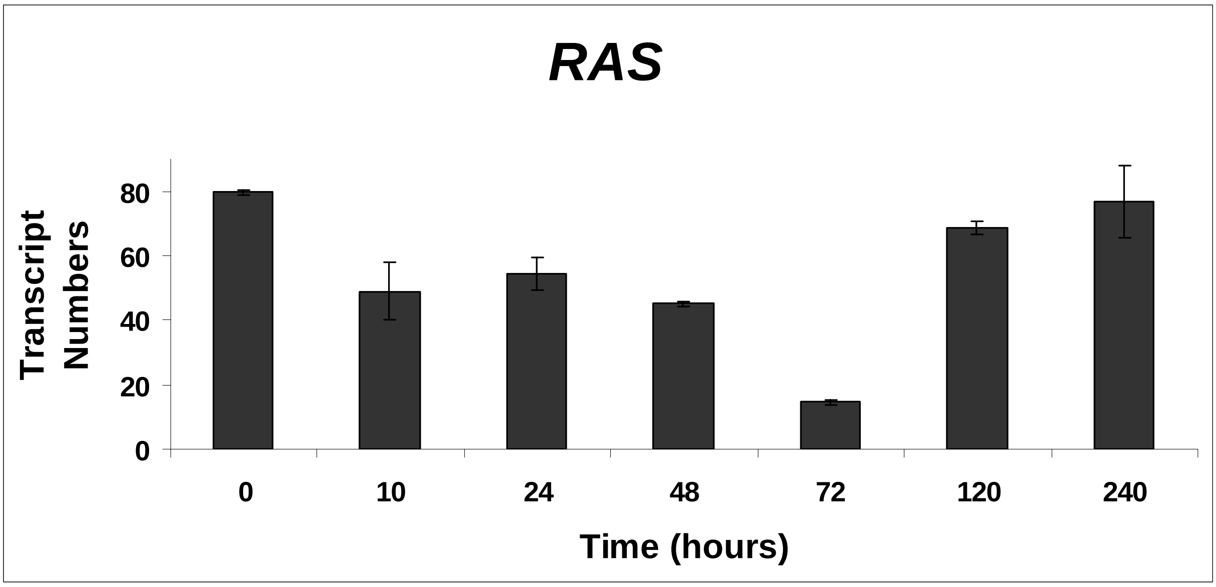

Supplement: Fig. S1 — The phylogenetic relationship between Paracoccidioides brasiliensis and other fungal cAMP-signalling proteins – a neighbour-joining bootstrap tree is derived from the amino-acid sequence alignments of fungal (A) adenylate cyclase (AC) (B) Gb, (C) Gg and (D) Ga proteins, using the VectorNTi 6.0 align program (Informax). [file mmi0065-0761_s1.pdf]
